# Metatranscriptome analysis to unveil the molecular signatures of transcriptionally active pathogens associated with bovine mastitis

**DOI:** 10.3389/fvets.2025.1642351

**Published:** 2025-10-16

**Authors:** T. Naveenprasath, Badeer Hassan Ummat, Farha Tarique, Aakash Chawade, Sandeep Kushwaha

**Affiliations:** ^1^National Institute of Animal Biotechnology, Hyderabad, India; ^2^Regional Centre for Biotechnology (RCB), Faridabad, India; ^3^Swedish University of Agricultural Sciences, Alnarp, Sweden

**Keywords:** bovine mastitis, RNA-Seq, metatranscriptomics, virulent protein, AMR, *Pseudomonas*, *Stenotrophomonas*, *Comamonas*

## Abstract

Bovine mastitis, a multi-etiological disease, is driven by complex microbial consortia; however, the transcriptional activity of pathogens and their underlying molecular mechanisms remains insufficiently explored. To the best of our knowledge, no metatranscriptome study on bovine mastitis is available in the public domain that identifies transcriptionally active pathogens and their associated molecular signatures. In this study, an *in silico* metatranscriptomics approach is employed on publicly available bovine mastitis RNA sequencing (RNA-Seq) datasets to identify transcriptionally active pathogens and their gene expression signatures. The analysis of unmapped reads (those not mapped to the bovine genome) identified 25 transcriptionally active pathogenic genera, accounting for 8,995 sequences, approximately from 500 bacterial strains of different species. Major findings of the study includes: (I) list of emerging pathogens “*Pseudomonas*, *Stenotrophomonas*, *Comamonas*, and *Sphingomonas*” actively contributing to disease development alongside well-known pathogens; (II) expression profiling of 4,121 virulence proteins, 484 peptidases, 432 secretory proteins, and 74 antimicrobial resistance genes; (III) identification of numerous hypothetical proteins in *Staphylococcus* (112), *Mycoplasma* (69), and *Escherichia* (32), representing potential source for diagnostics and multi-epitope vaccine candidates; and (IV) negative correlations between beneficial bacteria (*Blautia*, *Bacillus*, *Lactobacillus*) and pathogenic species in microbial co-occurrence interaction networks, suggesting opportunities for microbiome-based therapeutic strategies to treat subclinical mastitis. This study demonstrated the advantages of the metatranscriptomics approach and publicly available dual RNA-Seq datasets in unraveling the complexity of polymicrobial infectious diseases.

## 1 Introduction

Bovine mastitis is a multi-etiological disease in dairy cattle, characterised by inflammation of the mammary gland. It affects animal health, milk production and quality and causes substantial economic losses to dairy farmers (1). Additionally, treating bovine mastitis significantly contributes to the bigger problem of antimicrobial resistance (AMR), particularly through the potential use and misuse of antibiotics in livestock production (2). Various culture-dependent and culture-independent high-throughput research studies have reported the association of hundreds of microbial species, including *Streptococcus agalactiae, Streptococcus uberis, Staphylococcus aureus, Streptococcus pyogenes, Streptococcus dysgalactiae, Trueperella pyogenes, Escherichia coli, Klebsiella pneumoniae, Klebsiella oxytoca, Enterobacter aerogenes*, and *Pasteurella* spp. 16S rRNA and whole metagenome shotgun sequencing (WMGS) have significantly contributed to the discovery of microbial species associated with bovine mastitis (3–6). A 16S sequencing-based study reported the top 10 genera of causative pathogens in the milk of mastitic quarters from 65 cows, and also reported less-known bacteria, such as *Sneathia sanguinegens* and *Listeria innocua*, which are difficult to identify using culture-based diagnostics (7). Another 16S study highlighted significant variation in taxonomic profiles among different udder quarters (healthy, mastitis-affected, and quarters with undetermined status) at the genus level (8). Similarly, another study investigated the impact of bacteria causing subclinical mastitis on the structure of the cow’s milk microbiome, reporting that *Firmicutes* and *Proteobacteria* were predominantly present in subclinical mastitis (9). Burakova et al. (10) investigated the relationship between milk microbiome composition and bovine mastitis before and after antibiotic treatment using 16S rRNA sequencing. This study linked the increased abundance of the genera *Hymenobacter* and *Lachnospiraceae NK4A136* group with the development of subclinical and clinical mastitis. In contrast, a reduced abundance of *Ralstonia*, *Lachnospiraceae NK3A20* group, *Acetitomaculum*, *Massilia*, and *Atopostipes* in mastitic milk was linked to their potential role in maintaining udder health. In the 16S sequencing, specific regions of rDNA are targeted to characterise the community composition. However, it has several major limitations, including low taxonomic resolution (primarily at the genus level), PCR bias, limited functional insight, and a lack of viability assessment of the bacterial community (11–13). To overcome these limitations, the WMGS approach was employed to gain a deeper understanding of the functional aspects of microbial species associated with bovine mastitis. A milk WGS metagenome study on healthy and clinical mastitis (CM) cows reported 363 unique bacterial species and strains in CM samples (4). Another metagenomic analysis was performed on the subclinical mastitis milk samples of Kankrej, Gir (*Bos indicus*), and crossbred (*Bos taurus × B. indicus*) animals. A total of 56 different species, with varying abundances, were detected in the subclinical mastitis milk samples (6). Recently, a longitudinal study on the udder microbiome of Norwegian Red dairy cows was conducted using a shotgun metagenomic approach to gain insights into pathogen-driven microbial adaptation and succession. This study revealed that samples with low somatic cell counts were enriched with beneficial genera, such as *Corynebacterium*, *Bradyrhizobium,* and *Lactococcus*, while *Staphylococcus* predominated in high somatic cell count milk samples (14). In WMGS, the whole stretch of DNA is sequenced to identify the presence of genes and associated bacteria. However, it fails to distinguish between transcriptionally active and dead bacteria, and has low real-time functional resolution at the species and gene levels. These limitations can be addressed through a metatranscriptomics approach.

The accurate identification of molecular weapons used by disease-associated pathogens against their hosts is critical for gaining insight into disease initiation and progression (15). RNA-Seq datasets of diseases contain extensive functional information on disease-related pathogens that are expressed simultaneously (16). However, host-centric studies often overlook the functional importance of microbial communities, such as in bovine mastitis. The proportion of opportunistic and commensal microbial populations in the udder tissue constantly shifts as mastitis progresses, which can be utilised to understand *in vivo* host-pathogen interactions. Therefore, publicly available bovine mastitis dual RNA-Seq studies have been analysed to identify transcriptionally active bacteria associated with bovine mastitis, elucidating their functional role in pathogenesis. We extracted the non-bovine, non-ribosomal sequenced reads and constructed *de novo* metatranscriptome assemblies (17, 18). The assembled transcripts were further annotated to reveal the taxonomy and functional profile of transcriptionally active pathogens associated with bovine mastitis.

## 2 Materials and methods

### 2.1 Selection of bovine mastitis transcriptome studies

A systematic approach was employed to identify publicly available bovine mastitis transcriptomics datasets from the NCBI Sequence Read Archive (SRA) database. A total of eight bovine mastitis studies combining five field (PRJEB43443, PRJNA544129, PRJNA627642, PRJNA551141, and PRJNA668296) and three cell lines studies (PRJNA778892, PRJNA556769, and PRJNA556769) were selected for this study. The NCBI bio project ID and study details for each project are listed in Supplementary Table S1. The three studies (PRJNA778892, PRJNA556769, and PRJNA591729) utilised the bovine mammary alveolar cell line (MACT), while one study (PRJNA668296) employed blood, two studies (PRJEB43443 and PRJNA544129) used milk, and two studies (PRJNA551141 and PRJNA627642) used mammary gland samples to generate RNA-Seq data. A brief information of each study including experimental conditions, sample sizes, sequencing platforms, and read statistics are provided in Supplementary Tables S2.

### 2.2 Quality check and control of sequence data

The quality of the RNA-Seq datasets from each project was assessed using FastQC v0.11.9 and pre-processed using FastP v0.23.2. using default parameters (19). The pre-processed RNA-Seq reads were aligned against the *Bos Taurus* ARS-UCD1.3 genome using STAR version 2.7.3a (20) with a maximum number of multiple alignments set to 10. Further, unmapped reads were extracted, and ribosomal reads were removed from unmapped reads using SortMeRNA version 2.1b (21) with default parameters. High-quality unmapped non-ribosomal reads were used to construct the metatranscriptome assembly.

### 2.3 Metatranscriptome assembly and annotation

The *de novo* metatranscriptome was constructed from non-ribosomal unmapped short reads using the Trinity version 2.9.0 software (22). Protein-coding sequences were identified and translated using TransDecoder v5.5.0 for ORF identification with a minimum amino acid length of 60. Only complete ORFs with start and stop codons were retained for analysis to ensure the reliability of the generated protein sequences. The abundance of each translated transcript was calculated by counting the number of mapped reads to each transcript using STAR v2.7.3a and featureCounts v2.0 (23), followed by DESeq2 normalisation. Transcripts longer than 60 amino acids and with an average read count of ≥3 were selected for redundancy removal, which was carried out by clustering the sequences at 90% identity using CD-HIT v4.8.1 (24). Further, clustered transcripts were annotated using NCBI BLAST v2.12.0 (25) against the UniProt UniRef100 database. Only the annotated proteins with sequence identity ≥75% and query coverage ≥60% with the reference sequence were selected for further taxonomy and functional assignment. Microbial species and functional information for each sequence were extracted from the description of hit sequences. Furthermore, species-to-kingdom links were traced through the UniProt taxonomy tree map. The project PRJNA668296 was omitted due to technical incompatibility with the downstream analysis.

A list of 102 genera was compiled from published metagenomics studies on bovine mastitis to identify mastitis pathogens in the assembled metatranscriptome. KEGG Orthology (KO) annotations for sequences were obtained from eggNOG-mapper version 2.1.12 (26) using emapper.py, which assigned KO terms to each protein sequence. KO enrichment was performed using the enrichKO function of MicrobiomeProfiler (version 1.15) R package (24) at a *p*-value cut-off of 0.05 and *q*-value (FDR) cut-off of 0.2. Benjamini & Hochberg method was applied for *p*-value correction. Additionally, gene set filter criteria with a minimum size of 10 and a maximum size of 500 were used to reduce noise and exclude overly broad or narrow categories. The annotation of hypothetical protein sequences was performed using the InterProScan database (27). VirulentPred 2.0 (28), and signalP 5.0 (29) were used to identify virulent and secretory proteins. Peptidase and AMR genes were identified using a BLAST similarity search against the above-mentioned criteria in Merops [27] and the Comprehensive Antibiotic Resistance Database (CARD) (30).

### 2.4 Microbial diversity analysis of metatranscriptome

Microbial abundance data were pre-processed in the R language (version 4.2.3) using the R packages tidyverse (version 2.0) and RColorBrewer (version 1.1.3) (31). Microbial diversity, species evenness and richness analysis were performed using the R package vegan version 2.7. A linear regression model was fitted between Shannon diversity and species richness using the lm function, with 95% confidence intervals. The ggplot2 version 3.5 was used for visualization. Non-parametric Spearman correlation was calculated using the R cor function from the taxonomic abundance matrix. A correlation threshold of ≥0.6 and a *p*-value of ≤ 0.05 was applied to reduce noise while capturing biologically meaningful co-occurrence patterns. A microbial co-occurrence interaction network was generated using the R package igraph (version 2.1.4).

## 3 Results

**Figure 1:**
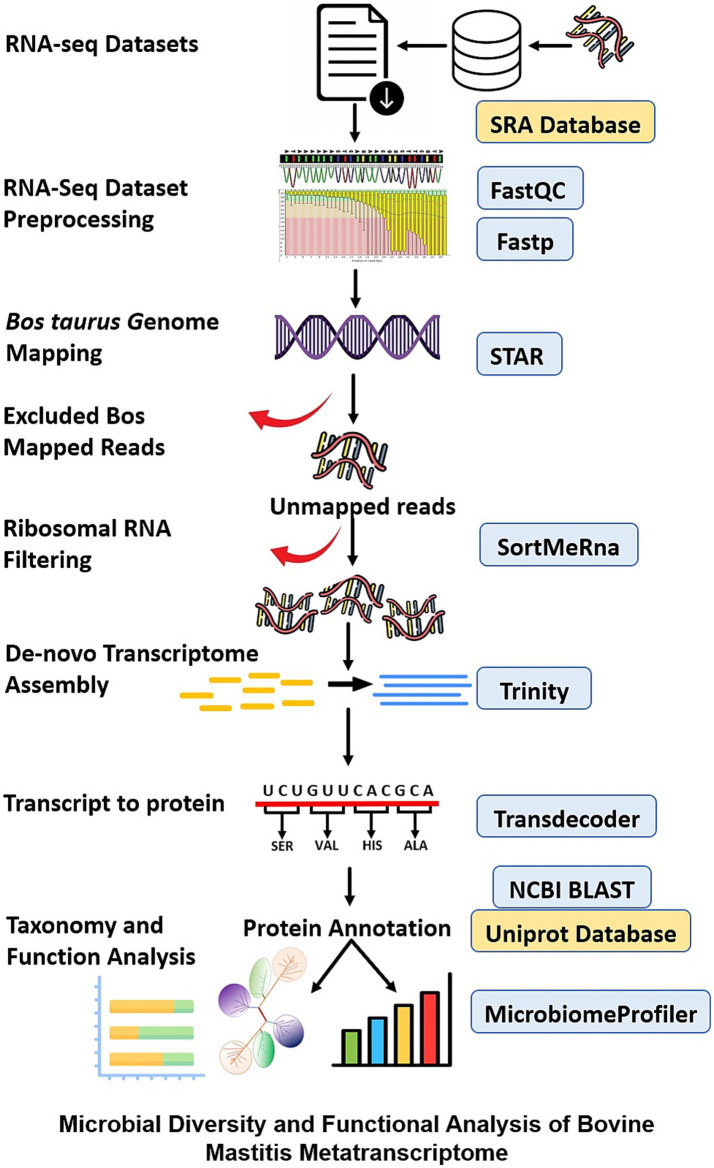
Schematic representation of the performed metatranscriptomics study to identify the transcriptionally active bacterial pathogens associated with bovine mastitis.

Host-centric RNA-Seq datasets are a valuable resource for exploring *in vivo* gene expression profiles of pathogens to develop new diagnostic and therapeutic solutions. In this study, non-ribosomal and unmapped reads from the host genome were extracted from all the selected RNA-Seq datasets and used for metatranscriptome assembly and analysis of pathogens. This study aims to identify transcriptionally active pathogens and their associated molecular signatures for bovine mastitis disease. The schematic workflow of the performed study is detailed in Figure 1.

### 3.1 Metatranscriptome assembly and annotation

The assembled metatranscriptomes were translated into protein sequences (Table 1). First, all transcripts were screened for an average read count of ≥3 and coded protein lengths of ≥60 amino acids to exclude chimeric and low-quality partial transcripts. Secondly, all the selected proteins at the first step were clustered at 90% sequence identity to remove redundant and similar proteins generated from different isoforms. In the third step, non-redundant protein sequences were annotated using a BLAST similarity search against the UniProt database. Only sequences that matched the UniProt database with at least 75% sequence identity and 60% query coverage were selected for downstream analysis, aiming to capture the inherent functional diversity of microbial consortia with greater accuracy. Microbial species information for each sequence was extracted from the description of hit sequences. Furthermore, species-to-kingdom links were traced through the UniProt taxonomy tree map. The above-mentioned filtering steps were applied to each project to ensure high-quality assembled transcripts and translated proteins for further taxonomic and functional analysis. The annotated protein sequences were classified into five groups based on their origin, namely bacteria, fungi, bovine, humans, and others (Table 2). This study primarily focused on identifying functionally active bacteria associated with bovine mastitis. Therefore, protein sequences from fungi, bovine, human, and other groups were removed after annotation from further downstream analysis.

**Table 1:** Summary of *de novo* metatranscriptome assembly for each RNA-Seq study.

Project	Data (million reads)	No of contigs	No of protein	Transcript filtering			
				Abundance >3 and length >60	Cluster at 90% identity	Blast annotation	Annotated proteins with identity >75 and coverage >60
PRJNA591729	99.57	394,208	161,589	5,183	3,036	2,977	2,442
PRJNA556769	22.37	148,064	80,312	9,908	6,337	6,178	4,869
PRJNA551141	11.99	86,899	29,603	13,222	12,682	12,368	10,431
PRJEB43443	174.58	378,422	246,442	67,401	41,220	39,417	25,577
PRJNA544129	40.6	47,042	27,901	20,573	18,661	17,673	1,582
PRJNA627642	5.15	7,517	4,520	3,701	3,282	3,051	1,174
PRJNA778892	2.34	57,039	36,470	21,077	19,868	19,183	12,986

**Table 2:** Summary of annotated metatranscriptome for each RNA-Seq study with the number of transcripts identified for bacteria, fungi, bovine, human and other categories.

Project	Annotated proteins with identity >75 and coverage >60	Source of sequences				
		Bacterial	Fungal	Bovine	Human	Other
PRJNA591729	2,442	250	0	1,318	9	865
PRJNA556769	4,869	44	0	3,263	23	1,539
PRJNA551141	10,431	7,895	6	516	43	1977
PRJEB43443	25,577	603	58	8,776	1,575	14,623
PRJNA544129	1,582	20	0	699	32	831
PRJNA627642	1,174	6	0	675	7	486
PRJNA778892	12,986	177	5	391	208	12,210

### 3.2 Metatranscriptome analysis to reveal taxonomic and functional profiling of pathogenic microbial communities associated with bovine mastitis

**Figure 2:**
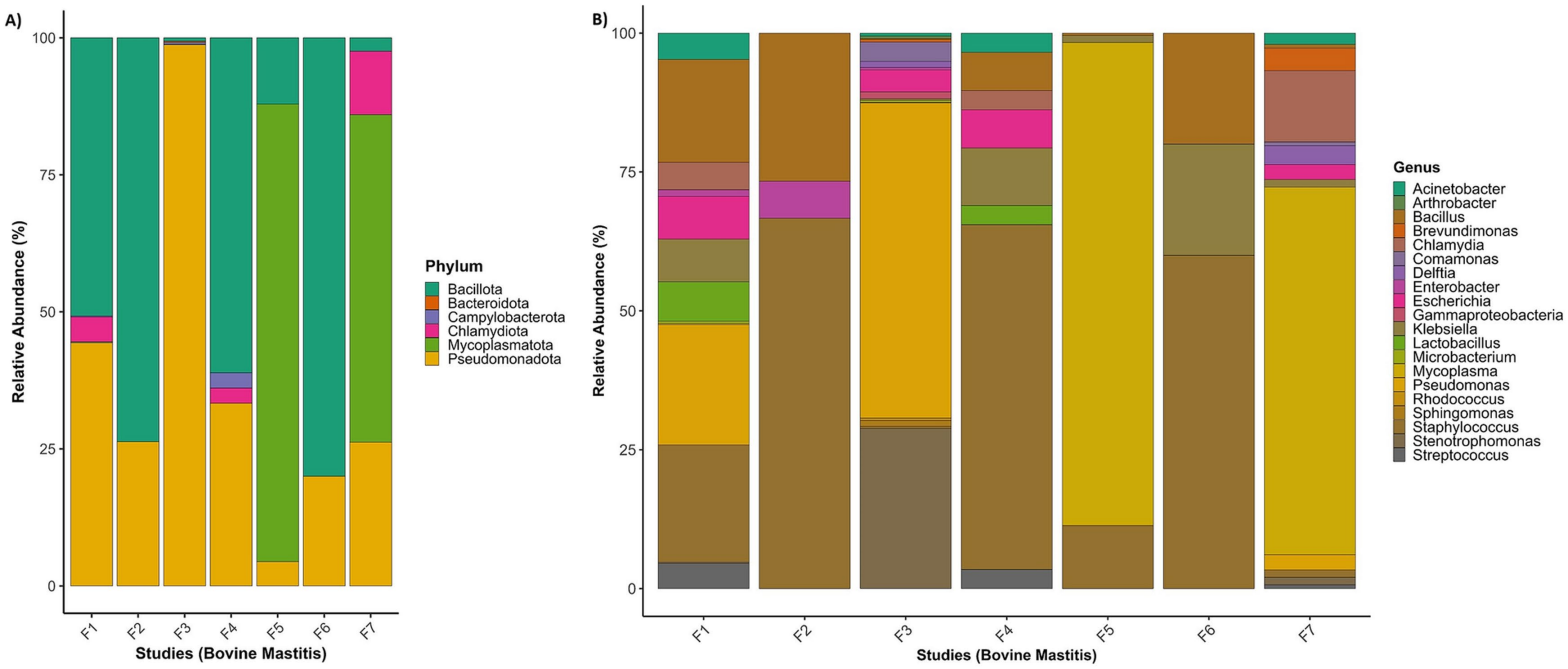
Taxonomic summary of metatranscriptome for each RNA-Seq study: **(A)** relative abundance of bacterial phyla, and **(B)** relative abundance of bacterial genera. F1 (PRJNA544129); F2 (PRJEN43443); F3 (PRJNA551141); F4 (PRJNA556769); F5 (PRJNA591729); F6 (PRJNA627642); F7 (PRJNA778892).

Protein sequences of bacterial origin were further analysed for each project to determine their taxonomic and functional role. Bacterial sequences were detected in distinct proteins of each project at the phylum, genus and species levels (Figures 2A,B).

However, various sequences were missing species-level information because the UniProt database may not contains species level information for these sequences. The phyla *Pseudomonadota* and *Bacillota* were highly prevalent in all projects and had a higher number of distinct sequences. Both phyla cover 16 to 96% of each project, with an average coverage of 70% (Figure 2). *Pseudomonas* is a highly abundant genus in the phylum *Pseudomonadota. P. alcaligenes, P. entomophila, P. marginalis, P. monteilii, P. phage, P. plecoglossicida, P. stutzeri, P. taiwanensis, P. geniculata, P. coronafaciens, P. savastanoi, P. amygdali, P. fluorescens, P. aeruginosa, P. syringae, P. putida* were among the most abundant species in the metatranscriptome. In the phylum *Bacillota*, various *Bacillus* species (*B. amyloliquefaciens*, *B. cereus*, *B. obstructivus*, *B. pumilus*, *B. subtilis*, *B. thuringiensis*, and B. wiedmannii) were also found in abundance.

In three projects, *Staphylococcus aureus* was used as *a* pathogen to study bovine mastitis infection, while the project PRJNA544129 studied naturally infected bovine mastitis. *Mycoplasma bovis* and *Streptococcus uberis* are the other two infection agents used in the projects PRJNA551141 and PRJNA627642, respectively. Table 3 shows that *Staphylococcus species (S. aureus, S. cohnii, S. sciuri, S. succinus)* have a significant number of distinct proteins in four projects. Project-wise identified genera, number of sequences and respective species are provided in Supplementary File 2 (ST1–ST8). Based on the number of sequences (≥5 distinct sequences), 58 transcriptionally active genera were identified. Transcriptionally active pathogenic genera and their top 20 species, with the number of distinct sequences, are provided in Supplementary File 2 (ST9 and ST10).

**Table 3:** Summary of identified bacterial species and number of bacterial proteins associated with species for each study.

Project	PRJNA 591729	PRJNA77 8,892	PRJNA 551141	PRJEB 43443	PRJNA 556769	PRJNA 544129	PRJNA 627642
Infectious agent	*Staphylococcus aureus*	*Staphylococcus aureus*	*Mycoplasma bovis*	*Staphylococcus aureus*	*Escherichia coli*	*Naturally infected*	*Streptococcus uberis*
Samples	*Cell line (MACT)*	*Cell line (MACT)*	*Mammary gland*	*Milk*	*Cell line (MACT)*	*Milk*	*Mammary gland*
Proteins	250	177	7,895	603	44	20	6
Species with the highest number of proteins	*Mycoplasma bovis, Staphylococcus aureus, Mycoplasma bovis DSM 22781, Mycoplasma bovis 1,067, Mycoplasma bovis 8,790, Mycoplasma agalactiae, Vibrio cholera, Klebsiella pneumoniae, Anaplasma phagocytophilum,*	*Mycoplasma hyorhinis, Mycoplasma hyorhinis HUB-1, Chlamydia trachomatis, Chlamydia abortus, Mycoplasma hyorhinis SK76, Escherichia coli, Brevundimonas* sp.*, Delftia tsuruhatensis, Bradyrhizobiaceae bacterium PARB1, Achromobacter* sp. *2789STDY5608621*	*Stenotrophomonas maltophilia, Pseudomonas* sp. *GM84, Pseudomonas putida, Pseudomonas aeruginosa, Pseudomonas plecoglossicida NB2011, Escherichia coli, Comamonas aquatic, Pseudomonas plecoglossicida, Stenotrophomona*sp.	*Staphylococcus aureus, Klebsiella pneumoniae, Escherichia coli, Acinetobacter baumannii, Lactobacillus johnsonii, Streptococcus pneumoniae, Bacillus subtilis, Pseudomonas* sp.*, Mycobacterium tuberculosis, Pseudomonas fluorescens*	*Staphylococcus aureus, Photobacterium ganghwense, Klebsiella pneumoniae, Mycoplasma arginine, Escherichia coli, Anaplasma phagocytophilum, Vibrio parahaemolyticus, Streptococcus pneumoniae, Salmonella enterica,*	*Staphylococcus aureus, Bacillus obstructivus, Bacillus cereus R309803, Anaplasma phagocytophilum, Porphyromonas macacae, Meiothermus silvanus, Enterobacter cloacae, Blautia luti,*	*Staphylococcus aureus, Nesterenkonia* sp. *M8, Klebsiella pneumoniae, Bacillus wiedmannii*

**Figure 3:**
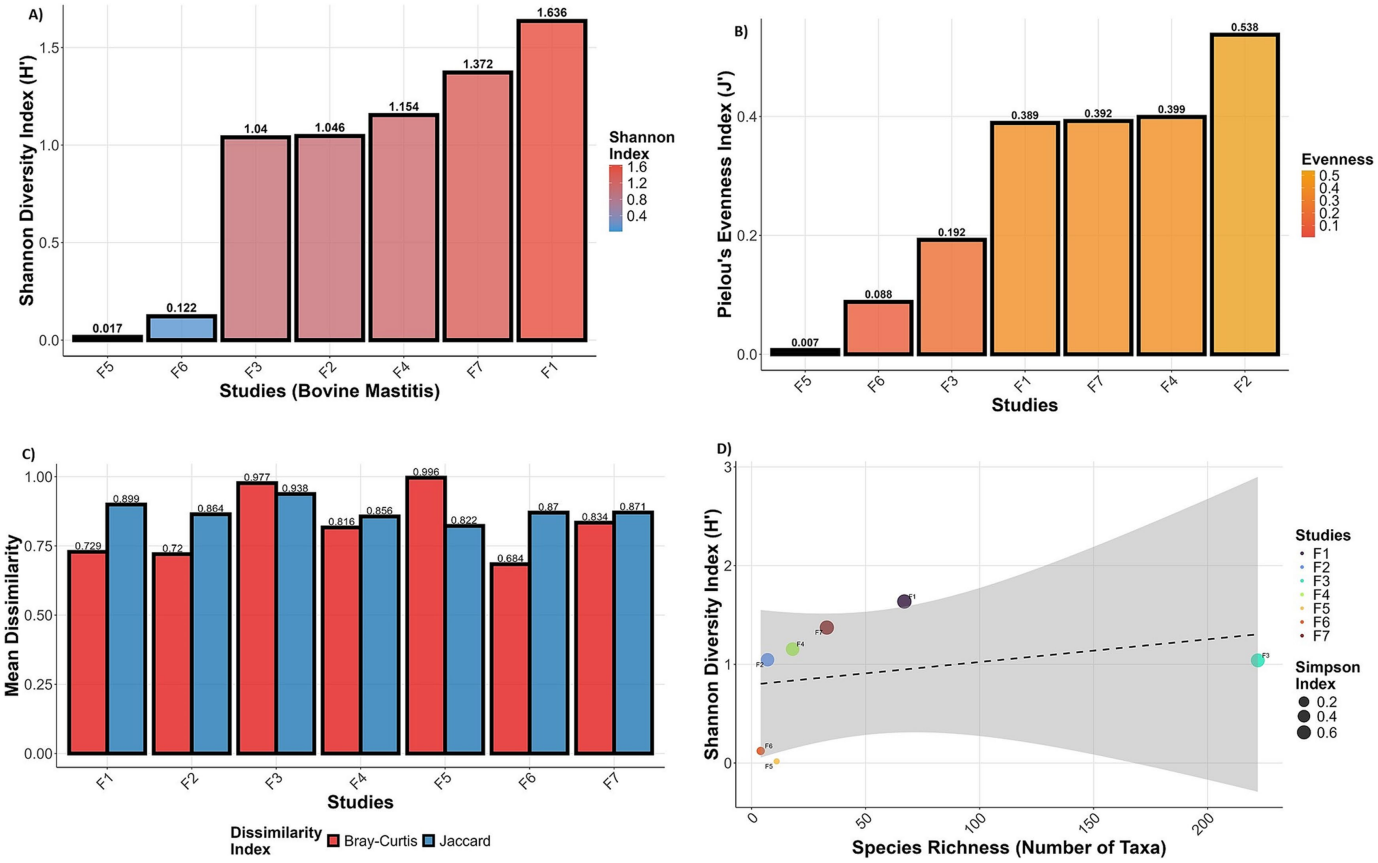
Microbial diversity indices of metatranscriptome for each RNA-Seq study: **(A)** Shannon diversity, **(B)** species evenness, **(C)** Bray–Curtis and Jaccard distance between samples, and **(D)** relationship between species richness and diversity. Box plots of Shannon, Simpson, Chao1, and Beta diversity for each project are provided in Supplementary Figures S1A–E. F1 (PRJNA544129); F2 (PRJEN43443); F3(PRJNA551141); F4(PRJNA556769); F5(PRJNA591729); F6(PRJNA627642); F7 (PRJNA778892).

The Shannon index of studies (PRJNA544129:1.63 and PRJNA778892: 1.37) exhibits moderate diversity, indicating the dominance of certain species, while others show lower diversity (dominance of a very few species) (Figure 3A). The diversity results are very well supported by the species evenness index. The studies (PRJNA591729, PRJNA627642, and PRJNA551141) have extremely uneven species composition, whereas PRJNA544129, PRJNA778892, and PRJNA556769 have low species evenness (Figure 3B). PRJEN43443 has moderate species evenness (0.5) among all the studies. The Bray-Curtis and Jaccard dissimilarity indices of all the studies are relatively high (>0.6), indicating high dissimilarity in microbial composition and abundance across all the studies (Figure 3C).

Each RNA-Seq study was designed differently according to its objectives. Therefore, we selected only mammary gland and milk samples and grouped them into ‘Mastitic’ and ‘Healthy’ groups to identify the most abundantly expressed proteins by calculating the mean abundance of their transcript sequences for each project separately. In study PRJEB43443, a time-point-based infection study, samples from the zero-hour time point were considered healthy, and all others were grouped as mastitic. Since PRJNA627642 does not have control samples, it was excluded from the abundance calculation. *Staphylococcus aureus* is the most prevalent pathogen of bovine mastitis reported in studies worldwide, and we also observed that *Staphylococcus aureus* proteins were among the most abundant proteins in all the projects. In our analysis, other known mastitis pathogens, such as *Escherichia, Mycoplasma, Streptococcus* and *Pseudomonas* species, were also present in higher abundance. We have observed that various transcripts exhibit higher average expression levels in mastitis samples. The proteins were sorted according to their abundance in the mastitis samples and provided the top five most abundant proteins across all projects (Table 4), which are primarily associated with cell division and gene regulatory mechanisms. Interestingly, most of the top-abundant proteins were either uncharacterised or hypothetical, with limited information available regarding their function. The project-wise average abundance of pathogens, as determined by identified distinct sequences, and the abundance of all identified sequences, are provided in Supplementary Table S3, Supplementary Files 1 and Supplementary Files 3a,b.

**Table 4:** List of the five most abundant proteins and their average abundance between two groups for each project.

Protein sequences	Abundance	
	Healthy	Mastitic
PRJNA591729		
Cell division protein FtsZ *n* = 1 Tax = *Mycoplasma bovis* TaxID = 28,903 RepID = A0A2N8U2C8_MYCBV	24635.4	14500.1
Chaperone protein DnaK *n* = 2 Tax = *Mycoplasma bovis* TaxID = 28,903 RepID = A0A454APB5_MYCBG	9926.2	17579.9
Cell division protein FtsZ *n* = 2 Tax = *Mycoplasma bovis* TaxID = 28,903 RepID = A0A454APL1_MYCBG	35500.5	20048.0
Transcriptional regulator MraZ *n* = 3 Tax = *Mycoplasma agalactiae* TaxID = 2,110 RepID = A5IYG3_MYCAP	173993.9	107345.4
Cell division protein FtsZ *n* = 2 Tax = *Mycoplasma bovis* TaxID = 28,903 RepID = A0A059XZB6_MYCBV	224068.3	125845.4
PRJNA551141		
Uncharacterized protein (fragment) *n* = 1 Tax = *Flavobacteriaceae bacterium* BH-SD17 TaxID = 2,487,930 RepID = A0A3N4N944_9FLAO	4455.7	5465.0
Uncharacterized protein (Fragment) *n* = 1 Tax = *Flavobacteriaceae bacterium* BH-SD17 TaxID = 2,487,930 RepID = A0A3N4N944_9FLAO	2870.2	7491.8
Uncharacterized protein (Fragment) *n* = 1 Tax = *Flavobacteriaceae bacterium* BH-SD17 TaxID = 2,487,930 RepID = A0A3N4N944_9FLAO	18560.8	26590.4
Uncharacterized protein (Fragment) *n* = 1 Tax = *Flavobacteriaceae bacterium* BH-SD17 TaxID = 2,487,930 RepID = A0A3N4N944_9FLAO	23650.0	43142.1
Hypothetical protein *n* = 1 Tax = *Vibrio cholerae* TaxID = 666 RepID=UPI000C288509	60705.1	68035.2
PRJEB43443		
Uncharacterized protein *n* = 2 Tax = *Anaplasma phagocytophilum* TaxID = 948 RepID = A0A181ZYN8_ANAPH	59.7	182.4
Hypothetical protein *n* = 1 Tax = *Xanthomonas oryzae* TaxID = 347 RepID=UPI000CA06AA2	1083.1	232.7
Hypothetical protein *n* = 1 Tax = *Staphylococcus aureus* TaxID = 1,280 RepID=UPI000E3C86C6	52.2	372.6
Hypothetical protein *n* = 2 Tax = *Staphylococcus aureus* TaxID = 1,280 RepID=UPI001156E9BB	346.0	1643.4
Hypothetical protein *n* = 1 Tax = *Staphylococcus aureus* TaxID = 1,280 RepID=UPI000E3E9016	653.2	1930.8
PRJNA544129		
Hypothetical protein *n* = 1 Tax = *Porphyromonas macacae* TaxID = 28,115 RepID=UPI000347688B	438.9	60.1
Hypothetical protein *n* = 1 Tax = *Staphylococcus aureus* TaxID = 1,280 RepID=UPI000EC6EB46	107.1	126.2
Reverse transcriptase family protein *n* = 1 Tax = *Staphylococcus aureus* TaxID = 1,280 RepID=UPI00115943A6	254.8	270.2
Endonuclease *n* = 1 Tax = *Staphylococcus aureus* TaxID = 1,280 RepID=UPI000E3CED8D	333.8	573.1
Hypothetical protein *n* = 1 Tax = *Staphylococcus aureus* TaxID = 1,280 RepID=UPI000E3E9016	1472.8	1830.6
PRJNA778892		
Hypothetical protein *n* = 1 Tax = *Escherichia coli* TaxID = 562 RepID=UPI0011AE3841	1.7	37.1
Dihydrolipoamide dehydrogenase of branched-chain alpha-keto acid dehydrogenase *n* = 1 Tax = *Mycoplasma hyorhinis* SK76 TaxID = 1,118,964 RepID=K7X8N3_MYCHR	5.2	39.5
Uncharacterized protein *n* = 2 Tax = *Anaplasma phagocytophilum* TaxID = 948 RepID = A0A181ZYN8_ANAPH	125.6	63.8
Adenine phosphoribosyltransferase *n* = 3 Tax = *Mycoplasma hyorhinis* TaxID = 2,100 RepID = A0A3B0PK95_MYCHR	1314.0	324.7
Hypothetical protein *n* = 2 Tax = *Staphylococcus aureus* TaxID = 1,280 RepID=UPI001156E9BB	697.6	350.2
PRJNA556769		
Protein A* *n* = 1 Tax = *Salmonella enterica* TaxID = 28,901 RepID=UPI000775F056	11.0	11.4
Uncharacterized protein n = 1 Tax = *Anaplasma phagocytophilum* TaxID = 948 RepID = A0A181ZWJ0_ANAPH	11.0	20.5
Endonuclease *n* = 1 Tax = *Staphylococcus aureus* TaxID = 1,280 RepID=UPI000E3CB2D5	83.5	81.4
Uncharacterized protein *n* = 2 Tax = *Anaplasma phagocytophilum* TaxID = 948 RepID = A0A181ZYN8_ANAPH	119.7	140.9
Hypothetical protein *n* = 2 Tax = *Staphylococcus aureus* TaxID = 1,280 RepID=UPI001156E9BB	190.5	274.4

### 3.3 KEGG orthology (KO) enrichment analysis of metatranscriptome

**Figure 4:**
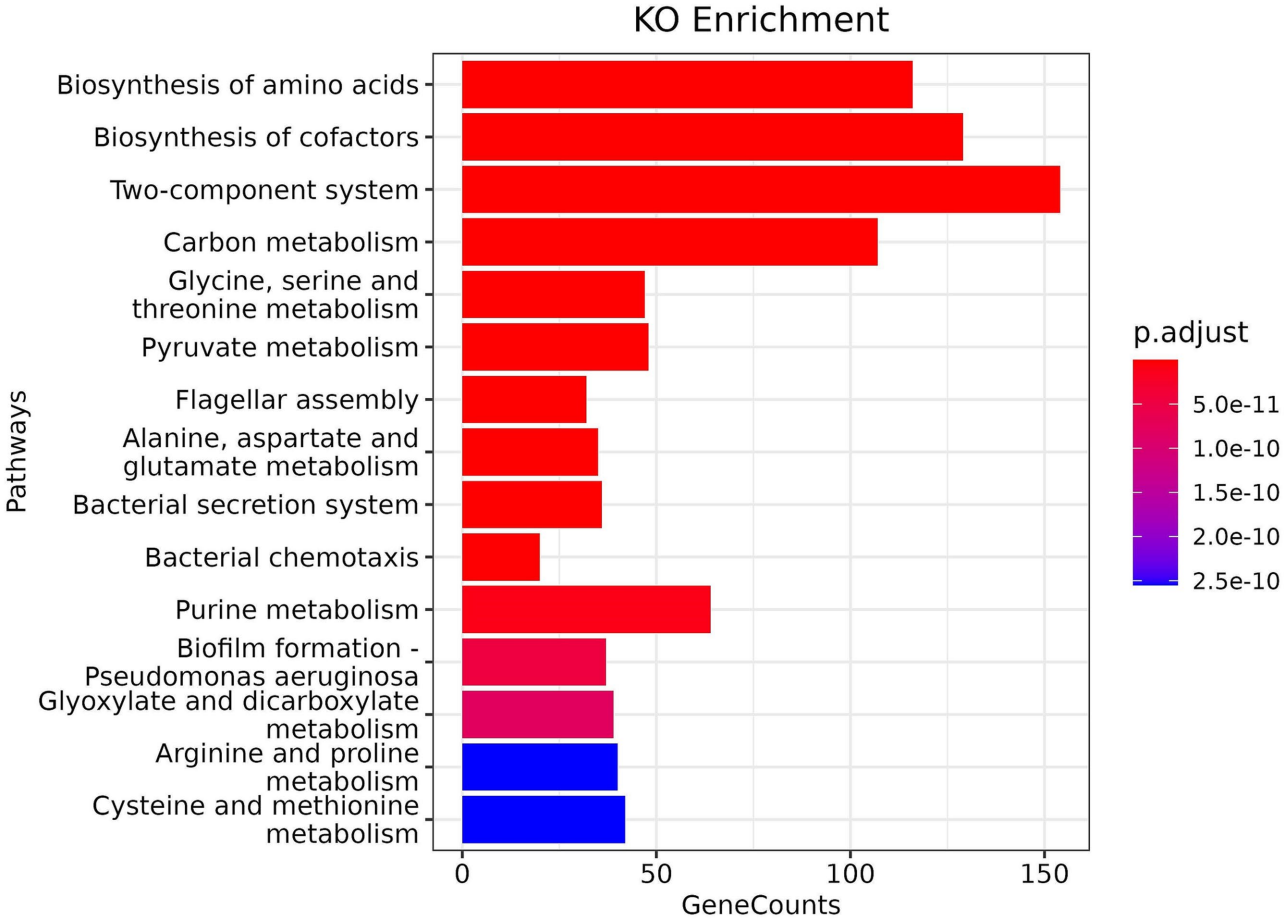
KEGG orthology (KO) enrichment analysis of assembled metatranscriptome.

KO enrichment analysis of the metatranscriptome is crucial for developing deep insight into bacterial pathogenesis, as enriched pathways can reveal key virulence factors, survival mechanisms, and employed metabolic adaptations in host environments. Overall, 83 pathways were obtained from KO enrichment. The description of pathways and counts of mapped KO terms is provided in Supplementary Table S5, Supplementary Files 1. Our KO analysis shows (Figure 4) that mastitis-associated pathogens are finely tuned to the host environment with a strong representation of signalling and regulation pathways (Two-component systems), nutrient acquisition and metabolism (amino acid and cofactor biosynthesis), motility and colonisation (flagella, chemotaxis), virulence and immune evasion (secretion systems, biofilm formation).

The two-component system, the most enriched pathway, enables bacteria to sense and respond to environmental changes, such as host defences and antibiotic treatment. The biosynthesis of amino acids and cofactors is another enriched pathway that suggests the survival and growth of pathogens in nutrient-limited host environments, activating pyruvate and amino-acid metabolism (including pyruvate, glycine, serine, and threonine) in response to stress. Flagellar assembly and chemotaxis pathways suggest bacterial motility for initial colonisation and host tissue invasion, and deliver effector proteins into host cells using bacterial secretion systems type III and VI secretion systems, a well-known virulence mechanism of bacteria. Biofilm formation pathways provide shelter against antimicrobials and host immunity, contributing to recurrence, chronic infections, and antimicrobial resistance. The enrichment of arginine, proline, cysteine, and methionine metabolism suggests that the pathogenic bacterial community has developed adaptive mechanisms (redox balance, stress response and polyamine synthesis) against host oxidative stress and immune modulation.

### 3.4 Identification of virulence factors, peptidase and AMR genes in metatranscriptome assembly

In bacteria, various types of proteins exist which can interact with the host directly or indirectly and may contribute to disease pathogenesis. Therefore, identifying such bacterial proteins in the metatranscriptome is essential for evaluating the pathogenic potential of pathogens. Virulent, peptidase and AMR genes fall under these categories. Therefore, bacterial proteins with virulence features were identified using VirlentPred software. Further, the predicted proteins were also explored in the Virulence Factor Database (VFDB) using BLAST similarity search (Table 5). Detailed descriptions of virulent proteins are provided in Supplementary File 4 (ST2). Predicted virulent proteins from *Stenotrophomonas, Mycoplasma, Staphylococcus Species, Comamonas,* and *Sphingomonas* species mostly belong to hypothetical and uncharacterized proteins.

**Table 5:** Summary of identified virulence protein, most abundant bacterial species, and associated virulence genes.

Project	PRJNA 551141	PRJEB 43443	PRJNA 778892	PRJNA 591729	PRJNA 556769	PRJNA 544129	PRJNA 627642
Identified virulent proteins	4,011	358	90	156	30	16	5
Most abundant bacterial species among identified virulent proteins	Pseudomonas species: *P. putida, P.* sp. GM84, *P. aeruginosa, P. plecoglossicida, P.* sp. NBRC 111124/111131*, P. mosselii, P. monteilii, P. fluorescens, P. geniculate*						
	Stenotrophomonas species: *S. maltophilia, S. maltophilia* SKK35/CC120222-04/CC120223-11						
	Mycoplasma species: *M. bovis, M. hyorhinis, M. bovis* (ATCC 25523 / DSM 22781 / NCTC 10131 / PG45)						
	Staphylococcus species: *S. aureus, S. succinus, S. sciuri*						
	Escherichia species: *E. coli, E. coli* CFT073, *E. coli* NA114, *E. coli*, UMNK88/MS 85/ISC7						
	Comamonas species: *C. aquatica, C. aquatica* st. DA1877*, C. testosterone, C. kerstersii*						
	Sphingomonas species: S. sp. IBVSS2, LK11, FARSPH, S. taxi, *S. sanguinis*						
	Delftia species: *D. tsuruhatensis, D. acidovorans, D. lacustris, Delftia* sp. ZNC0008						
Virulence Genes associated with bacterial species	Pseudomonas species: PA0685, PA0686, PA0687, PA1458, PA1459, PA1464, PA14_RS24370, PA1663, PA3349, PA4706, PFL_RS24295, PSEEN_RS11600, PSPTO_RS07240, alg(44,8, B, C, F, K, L, W, X, Z), cheZ,chp(A, C), clpV1, crc, cup(A3, B5), estA, exlA, fap(D, F), fimV, fle(N, Q, R, S), flg(A, C, D, E, F, H, I, J, K, L, M),flh(A, F), fli(C, G, H, I, J, K, M, R), fpv(A, R), gacS, hasE, hcnB, hdtS, hopAJ2, hsi(G1, J1), mot(B, C, D, Y),muc(A, B, D, P), pch(A, E), pcrD, phuR, phuT, phz(D1, H), pil(C, J, M, Q, R, T), pprA, prpL, pvd(A, D, E, H, I, J, L, M, N, O, Q, Y), rpoN, tadC, tagS, tke4, tss(B1, F1, H, K, L1), vfr, vgrG1a, waa(A, C, F, G, P), xcp(Q, R, Y)						
	Klebsiella species: KP1_RS17295, KPHS_35550, KPR_RS09000, acr(A, B), allC, clpV, gndA, impH, ugd, vgrG,						
	Escherichia species: ECNA114_RS14835, cah, cheB, f17d-C, fepA, flhB, iroN, ompA, tar, tssH, vgrG						
	Mycobacterium species: devR/dosR, glcB, glnA1, katG, mgtC, narG, purC,						
	Acinobacter species: adeG, bfmR, gsp(E2, F), pilC						

Proteases are key bacterial proteins that cleave peptide bonds in proteins to facilitate host cell colonisation, defence evasion, and damage. The cross-compatibility of several bacterial proteases with the host system enables them to cleave host proteins and disrupt host cellular functions. MEROPS database was used to identify the peptidase sequences among bacterial proteins (Table 6). The identified peptidases mainly belong to the serine and metalloprotease families, and the PRJNA551141 project has the highest number of peptidases. Proteases were identified for the following projects: PRJNA551141 (625), PRJEB43443 (32), PRJNA778892 (26), PRJNA591729 (9), and PRJNA556769 (4). Project-wise information on identified proteases is provided in Supplementary File 4 (ST3). The prevalence of antibiotic resistance among bacterial pathogens is increasing due to the unsupervised use of antibiotics in treatment, and the treatment of bovine mastitis contributes significantly to antimicrobial resistance. Therefore, the identified bacterial proteins from each project were analysed against the CARD database (Table 7). A total of 85 proteins were predicted to have antibiotic resistance; 82 sequences belonged to study PRJNA551141. Two resistance proteins were identified from PRJEB43443 and PRJNA778892, respectively. The identified proteins from the bovine mastitis metatranscriptome are resistant to major antibiotics and drugs, such as Tetracycline, Penicillin, Macrolides, and Cephalosporins, which are routinely used for bovine mastitis treatments. A project-wise detailed description of antibiotic resistance proteins is provided in Supplementary File 4 (ST4).

**Table 6:** Summary of identified protease gene families, most abundant bacterial species, and associated protease families in metatranscriptome.

Analysis	Description
Total number of peptidase proteins	721
The most abundant peptidase family and count	Serine peptidases family: S9 (46), S12 (16), S16 (12), S33 (43)
	Cysteine peptidases family: C26 (20), C44 (12), C56 (11)
	Metallo peptidases family: M13 (10), M38 (40), Subfamily M23B (13)
	Unknown type peptidases family U69 (21)
	Inhibitor proteases family: I87 (20)
	Aspartic peptidases family: A2 (10)
The most abundant peptidase protein family and count	Stenotrophomonas species: (*S. maltophilia, S. maltophilia* sp. CC120223-11, RIT309, SKA14) Peptidases families: A24, C14, C26, C40, C44, C56, C59, C82, G05, I39, I78, I87, M01, M02, M03, M103, M13, M14, M15, M16, M19, M20, M23, M24, M28, M38, M48, M50, M56, M61, S01, S08, S09, S11, S12, S14, S15, S16, S33, S41, S45, S46, T01, T03, U69,
	Pseudomonas species: (*P. aeruginosa, P. asiatica, P. brassicacearum, P. capeferrum, P. entomophila, P. fluorescens, P. mendocina, P. monteilii, P. mosselii, P. plecoglossicida, P. putida, P. resinovorans, P. taiwanensis, P.* sp. 10-1B, 2_1_26, Ag1, B11-1, BAY1663, C5pp, GM55, GM84, NBRC 111131, PAMC 25886, TJI-51, TKP, VLB120) Peptidases families: A24, A39, C13, C26, C39, C40, C44, C45, C56, C82, G05, I13, I39, I42, I87, M01, M03, M103, M14, M15, M16, M17, M18, M19, M20, M23, M24, M38, M41, M42, M48, M50, M67, M75, M90, N04, S01, S08, S09, S11, S12, S13, S16, S24, S26, S33, S45, S49, S54, S66, T03, T05, U32, U69
	Escherichia species: (*E. coli, E. fergusonii, E. sp.* 4_1_40B, KTE114, KTE31, TW09308) Peptidases families: A26, C44, C51, C56, C82, G04, I39, M103, M17, M20, M24, M38, M74, N06, S11, S33, T02, U32, U69, X20,
	Mycoplasma species: (*M. agalactiae, M. bovis, M. hyorhinis*) Peptidase families: M03, M17, M26, M41, M42, S08, S41

**Table 7:** Summary of AMR genes and gene families, resistance mechanisms, most abundant bacterial species, and associated AMR genes in metatranscriptome.

Analysis	Description
Drug classes associated with resistance proteins	Tetracycline antibiotic (39), Macrolide antibiotic (35) Fluoroquinolone antibiotic (33), Phenicol antibiotic (31) Penam (28), Cephalosporin (23), Monobactam (21), Disinfecting agents and Antiseptics (20), Carbapenem (20), Aminocoumarin antibiotic (19), Penem (17), Diaminopyrimidine antibiotic (17), Cephamycin (17), Peptide antibiotic (15), Sulfonamide antibiotic (9), Aminoglycoside antibiotic (8), Glycylcycline (7), Nitrofuran antibiotic (4), Rifamycin antibiotic (2), Nucleoside antibiotic (1), Mupirocin-like antibiotic (1), Bicyclomycin-like antibiotic (1)
Bacterial species associated with resistance proteins	*Pseudomonas aeruginosa* PAO1 (27), *Pseudomonas aeruginosa* (16), *Escherichia coli* str. K-12 substr. MG1655 (10) / W3110 (5), *Escherichia coli* (4), *Escherichia coli* KTE14 (2), *Salmonella enterica* subsp. *enterica* serovar Typhimurium str. LT2 (4), *Stenotrophomonas maltophilia* (4), *Aeromonas caviae/salmonicida* (3), *Klebsiella pneumoniae* (3), *Acinetobacter baumannii* (2), *Nocardia farcinica* IFM 10152 (1), *Photobacterium damselae* subsp. *Piscicida* (1), *Staphylococcus aureus* subsp. *aureus* USA300_TCH959 (1), *Vibrio fluvialis* (1), *Bifidobacterium bifidum* PRL2010 (1),
Resistance protein’s mechanism of action and count	Antibiotic efflux (71), Antibiotic target alteration (7) Antibiotic inactivation (5) Reduced permeability to antibiotic (4) Antibiotic target replacement (2)
Most abundant species and their expressed AMR genes	*Pseudomonas aeruginosa*: APH(3′)-IIb, PDC-456, Mex(A, B, F, J, K, L, N, Q, W, Y), Mux(A, B, C), Opm(B, H), Opr(J, M), Paer(CpxR, soxR), ParR, Tri(A, B, C), basS, bcr-1 *Escherichia coli*: CRP, Ecol_acrA, Kpne_KpnH, PmrF, eptA, mdt(B, C, E, F, P),oqx (A, B), ugd, *Salmonella enterica*: mds(A, B) *Stenotrophomonas maltophilia*: sme(E, F) *Aeromonas caviae/salmonicida*: Mox-4, tet-C *Klebsiella pneumoniae*: Kpne_OmpK37, OmpA

**Figure 5:**
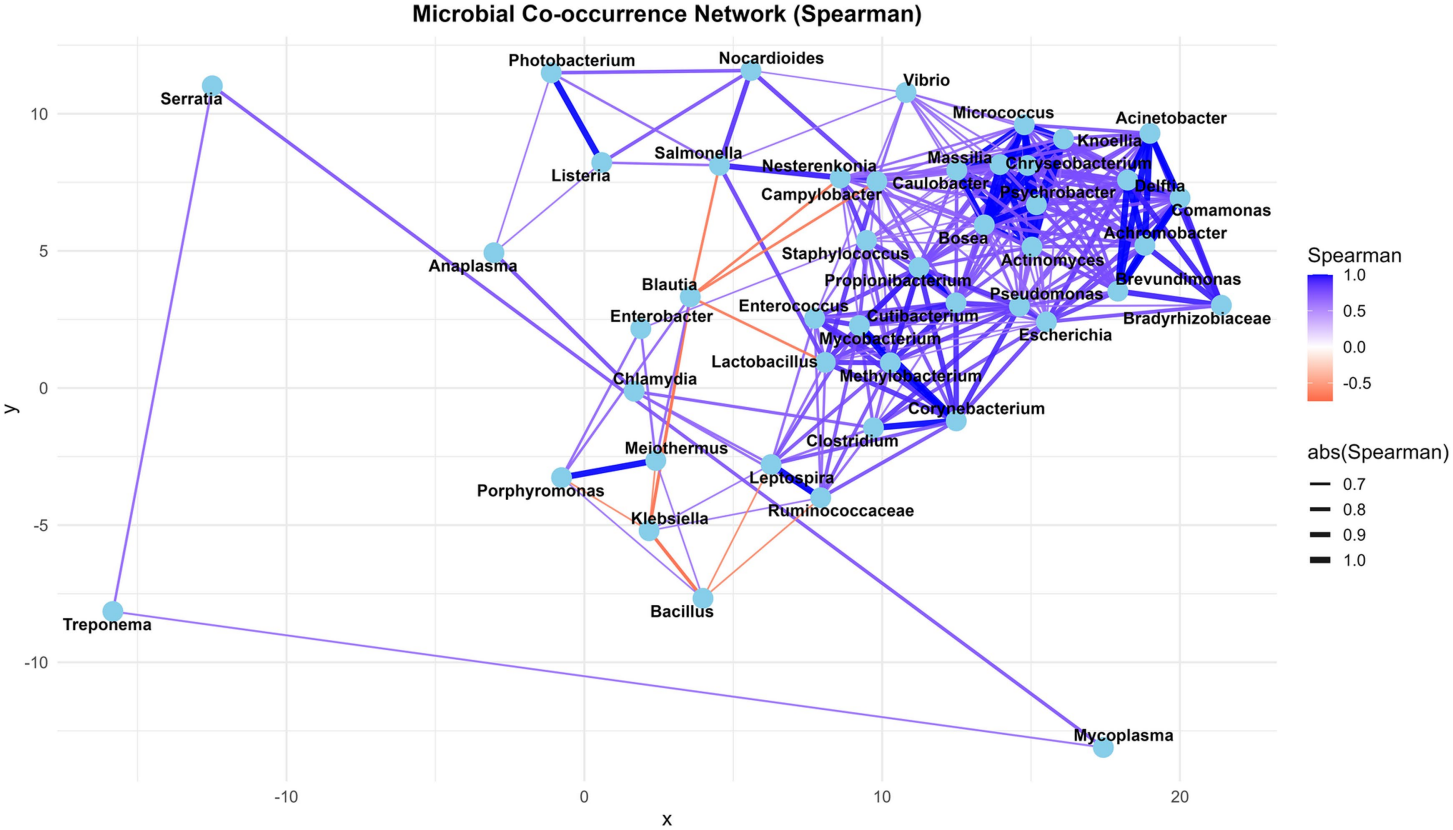
The microbial co-occurrence interaction networks of bovine mastitis-associated microbial communities at the genus level, based on the Spearman correlation coefficient.

Based on metatranscriptome analysis, a list of the 25 most transcriptionally active bacterial genera has been compiled to illustrate the active pathogenic load associated with bovine mastitis, with a particular focus on virulence factors, peptidases, secretory proteins, and antibiotic resistance (AMR) genes. The minimum number of virulent proteins, 10, was considered to select the most transcriptionally active genera from metatranscriptome data (Table 8). A detailed description is provided in Supplementary File 2 (ST11). Microbial co-occurrence interaction network analysis, based on expression abundance, revealed that *Blautia*, *Bacillus*, *Klebsiella*, and *Lactobacillus* are negatively correlated with other genera of pathogenic consortia. It suggests that the abundance of *Blautia and Lactobacillus*, a genus with probiotic characteristics, decreases during disease, whereas the genus *Bacillus* contains both pathogenic and probiotic species; perhaps the abundance of probiotics is more prevalent in healthy conditions. Interestingly, a significant number of virulent (33), peptidase (2), and antibiotic resistance (AMR) (3) proteins were identified in the *Klebsiella* genus from metatranscriptome data. However, it is negatively correlated to consortium species (Figure 5).

**Table 8:** List of transcriptionally active bacteria associated with bovine mastitis based on the number of virulent proteins identified for each genus.

Number of sequences identified from bovine mastitis metatranscriptome								
Genus	Seq	Sp/St.	Genes	Hyp/Un	Vir	Pept	Sec	AMR
*Pseudomonas*	4,261	205	3,898	363	1952	231	198	43
*Stenotrophomonas*	2,108	44	1892	216	1,226	149	170	4
*Mycoplasma*	312	13	243	69	175	14	35	0
*Staphylococcus*	191	5	79	112	132	1	0	1
*Escherichia*	335	17	302	33	106	48	7	21
*Comamonas*	257	8	237	20	93	13	7	0
*Sphingomonas*	80	23	62	18	51	4	1	0
*Delftia*	85	14	77	8	44	0	0	0
*Klebsiella*	67	6	36	31	42	2	0	3
*Bacillus*	114	38	72	42	37	10	2	0
*Chlamydia*	45	4	20	25	30	0	0	0
*Acinetobacter*	66	15	34	32	28	1	0	2
*Brevundimonas*	39	17	36	3	23	2	3	0
*Enterococcus*	28	3	2	26	23	0	0	0
*Microbacterium*	30	24	22	8	20	0	0	0
*Streptococcus*	28	5	19	9	20	0	0	0
*Arthrobacter*	34	5	32	2	17	0	0	0
*Caulobacter*	24	10	22	2	16	2	2	0
*Rhodococcus*	30	14	25	5	15	0	0	0
*Enterobacter*	38	9	28	10	13	3	0	0
*Campylobacter*	28	7	26	2	13	1	6	0
*Mycobacterium*	20	3	6	14	13	3	0	0
*Archangium*	16	1	10	6	12	0	0	0
*Anaplasma*	16	1	0	16	10	0	1	0
*Aquabacterium*	16	2	14	2	10	0	0	0

Seq, sequences; Sp/st, species/strains; Hyp/Un, hypothetical or uncharacterised; Vi, virulent; Pept, peptidase; Sec, secretory; AMR, antimicrobial resistance.

## 4 Discussion

This study primarily focused on identifying transcriptionally active bacteria from publicly available bovine mastitis dual RNA-Seq studies, as these datasets contain extensive functional information on disease-related microbial species within the same biological samples. The microbial content expressed during the host-pathogen interaction, provides *in vivo* gene expression profile of pathogens, which facilitates the discovery of bacterial virulence factors to develop new diagnostics and therapeutics. Similar attempts have been made to understand the role of microbial communities in human cancer diseases using RNA-Seq data (34, 35).

In this study, rigorous quality control criteria were used to exclude chimeric, low-quality, partial and redundant transcript sequences. A minimum protein length of ≥60 was used to include small proteins from microbial communities that are involved in regulating larger proteins, cell signaling, antibiotic production and toxin regulation, membrane-associated functions, metabolism, and stress response, as well as metal ion homeostasis (36). Moreover, the inclusion of 60 amino acids provided higher confidence in functional gene-to-species assignment compared to shorter lengths and relatively less partial sequences. The average read counts of greater than or equal to 3 for genes across the samples were considered to remove chimeric and rare protein sequences, due to the hypersensitivity of high-throughput sequencing technology. The average read count is a better metric for reducing disparity between small and large numbers of studies. Various isoforms of microbial gene sequences were generated due to the stitching of short k-mers during transcriptome assembly. Therefore, we clustered protein isoforms at 90% sequence identity to reduce functional redundancy and false specificity for cross-species orthology inference. Stringent BLAST similarity search criteria (sequence identity ≥75% and query coverage ≥60%) were used to ensure the high-quality taxonomical and functional assignment (Table 2). This approach is more reliable and accurate than the seed sequence mapping approach, which lacks robustness for assigning gene species links in microbial community analysis.

Even after excluding non-host reads before assembly, a large number of the proteins belonged to the bovine species, which may be due to the presence of conserved domains and motif sequences across the kingdom. In filtered protein sequences, the proportion of bovine sequences varied from 3% to 71%, compared to the low bacterial protein proportion (0.5%–10%) across all projects, except for study PRJNA551141. It has 75% bacterial protein and 5% bovine protein. The enhanced representation of bacterial proteins may be due to the fact that rRNA was removed during sample preparation, as rRNA constitutes 70%–90% of the total RNA in these samples. None of the other projects had used ribo-depletion during their sample preparation.

The microbial diversity, species richness and evenness metric support the functional diversity among the selected RNA-Seq studies. The scatter plot of species richness and Shannon diversity index shows a positive correlation between species richness and Shannon diversity, as indicated by the upward-sloping linear regression line (Figure 3D). This suggests that sample PRJEB43443 demonstrated a relatively high Shannon index with moderate species richness, potentially indicating a more evenly distributed community structure, as supported by its larger point size (Simpson index). In contrast, sample PRJNA551141, while showing the highest richness, exhibited only moderate Shannon diversity, suggesting dominance by a few taxa (*Stenotrophomonas* and *Pseudomonas* species) and reduced evenness. Higher or moderate evenness often suggests stable communities, while low evenness may indicate vulnerability to disturbance (PRJNA591729 and PRJNA627642). Low Shannon diversity and high species richness indicate dysbiosis or infection dominance (PRJNA551141). Moderate Shannon diversity and moderate species richness may reflect baseline microbial communities (PRJNA544129), whereas moderate diversity with fewer taxa suggests a balanced microbial environment with minimal colonisation (PRJNA778892), possibly post-antibiotic or in the early stage of infection.

In KO analysis, enriched pathways include the two-component system, amino acid/cofactor biosynthesis, flagellar assembly and secretion systems, and biofilm formation, reflecting bacterial adaptation to host defences, nutrient limitation, and persistence mechanisms. Enrichment of amino acid metabolism further highlights adaptive responses of the pathogenic community to oxidative stress and immune modulation (37–41). The Project-wise KO analysis is provided in Supplementary Figures S2A–E. Various highly abundant sequences were either uncharacterised or hypothetical despite their known genomic sequences (Table 4). Therefore, *in silico* sequence analysis of hypothetical and uncharacterised proteins was performed to explore their functional information. On average, 62% of the total putative proteins were annotated, which contain domains of catalytic enzymes, transposable elements, retrotransposable elements, reverse transcriptases, prokaryotic membrane lipoproteins, endonucleases, and various other molecular signatures. A summary of hypothetical protein annotation and detailed descriptions of the InterProScan annotation of all hypothetical proteins are provided in Supplementary Table S4 and Supplementary File 4 (ST1).

In our analysis, various abundant hypothetical and uncharacterised proteins contain intrinsically disordered regions, suggesting that these proteins may have roles in host–microbe interactions *in vivo* conditions, such as bacterial effector protein translocation, evasion of the host immune system, and mimicing host protein functions (42). The presence of intrinsically disordered regions in secreted bacterial effectors is well-reported (43). The biofilm-forming curli protein of *E. coli* has disordered regions that facilitate its aggregation and contribute to the formation of the extracellular matrix of the biofilm (33). Various intrinsically disordered regions containing hypothetical or uncharacterised proteins exhibit a virulent nature in our analysis. The *in silico* annotated sequence contains reverse transcriptases, transposable elements and transposon-related domains. Reverse transcriptase converts RNA into double-stranded cDNA, and it is a major component of diversity-generating retroelements (DGRs) in bacteria. Reverse transcriptases in DGRs can create hypervariable regions in target genes, fuelling the faster evolution of bacterial genomes to ensure bacterial survival in harsh environments (44). Transposable elements can also alter the genome by insertion and excision, promoting bacterial evolution. These jumping genes are known to be part of bacterial virulent elements (45). These proteins may have roles in antibiotic resistance and pathogenicity, which need to be explored further.

Based on the number of virulent proteins (≥10), peptidases, secretory proteins, and antibiotic resistance (AMR) genes, a list of 25 transcriptionally active bacterial genera was compiled to highlight pathogenic load associated with bovine mastitis. *Pseudomonas* species dominated in the constructed metatranscriptome with the highest number of species (205) and expressed genes (3898), including the highest number of virulence (1952), secretory (198), peptidases (231) and antimicrobial resistance genes (44), highlighting a significant role of *Pseudomonas* species in bovine mastitis pathogenesis. Moreover, *P. putida, and P. aeruginosa* are emerged as multidrug-resistant pathogens linked with clinical and subclinical bovine mastitis worldwide (46–49). *Stenotrophomonas* species, a lesser-known pathogen of bovine mastitis, have been shown to express substantial virulence genes (1226) and secretory proteins (170), indicating their active involvement in pathogenesis and potential biofilm formation or host interaction, making them an emerging global opportunistic pathogen (50). Recent research also demonstrated that enterogenic *S. maltophilia* can migrate from the gut to the mammary gland via the gut-mammary axis to induce mastitis by activating the calcium-ROS-AMPK-mTOR-autophagy pathway (51). *Mycoplasma* species, despite having a lower virulence gene count (175) out of a total of 243 identified genes, indicate a specialisation in pathogenesis rather than metabolic versatility. *Staphylococcus,* a known pathogen of mastitis, displayed a lower number of species but a notable number of virulence factors (132). The expression of hypothetical or uncharacterised sequences (112) suggests the presence of novel molecular factors from these species in bovine mastitis pathogenic consortia.

Among all identified virulent genes, 633 were uncharacterized, and 379 were hypothetical by Uniprot definition. The protein sequence (Sequence id: TRINITY_DN43764_c0_g1_i1.p4; Uniprot id of hit: UniRef100_UPI000DEBC0B7) from *Pseudomonas aeruginosa* is one of the identified virulent hypothetical proteins that also possesses an AMR gene feature (Drug class: disinfecting agents and antiseptics; mode of mechanism: Antibiotic efflux; ARO ID:3003680; Gene name: TriB). In our study, we identified 84 antimicrobial resistance (AMR) genes, some are reported for bovine mastitis, including tetG, tetC, blaZ, sul1, adeB, acrA, APH(3′)-IIb, mexA, mexB, and mexF. These genes are involved in well-known resistance mechanisms, including efflux pumps, antibiotic-modifying enzymes, and target protection proteins. Other genes like adeB, adeF, APH(3′)-IIb, eptA, kpnH, ompA, oqxA, and oqxB were also reported for bovine mastitis. The presence of these AMR genes in this dataset suggests a broader distribution of resistance determinants among mastitis-associated microbiota (52–54). The association of other identified AMR genes with bovine mastitis has not been found in the literature. In the absence of literature support, it is challenging to speculate on the role of these bacteria in pathogenic consortia mastitis, which are predominantly composed of *Pseudomonas* species. However, the expression of such a large number of AMR genes from *Pseudomonas* species suggests a potential role in providing protection against antibiotics, which warrants further in-depth investigation. Most *Pseudomonas aeruginosa* strains possess a Type III Secretion System (T3SS), which plays a crucial role in pathogenesis and has been linked to elevated somatic cell counts in the milk of cows with mastitis (55). Key T3SS-related genes identified in bovine mastitis isolates include *exlA*, *pcrD*, and *hopAJ2*. Additionally, a range of other virulence and secretion system genes—such as *clpV1*, *flgC*, *flgG*, *flgK*, *flgL*, *fliC*, *mucA*, *mucB*, *algD*, *algF*, *algA*, *pilV*, *pilY2*, *pvdF*, *pvdS*, *rhlR*, *rhlI*, *rhlA*, *rhlB*, *rhlC*, *tssB1*, *tssF1*, *tssG1*, *vgrG1*, as well as components of the *xcp* (PA3095–PA3105) and *hxc* (PA0677–PA0687) secretion system are regulated by phosphate availability (56).

The negative correlation of *Klebsiella* with other genera of pathogenic consortia (Bacillus/ *Balutia*/ *Salmonella*/ *Leptospira*/ *Porphyromonas*/ *Ruminococcaceae*/ *Lactobacillus*) in the non-directional microbial co-occurrence network indicates that when *Klebsiella* abundance increases, *Bacillus/ Balutia/ Salmonella/ Leptospira/ Porphyromonas/ Ruminococcaceae/ Lactobacillus* tends to decrease, and vice versa. This could be due to competition, niche exclusion, or antagonistic interactions. These species may not co-exist well in the same environment. Some species, such as *Bacillus/Lactobacillus*, produce bacteriocins that inhibit the growth of *Klebsiella* (57). Host factors (such as temperature, oxygen level, or pH) may favour the growth of *Klebsiella* species after environmental *E. coli* infection compared to other pathogens, due to similar growth conditions to those of other *Enterobacteriaceae*. The chance of *Klebsiella* infection after exposure to environmental mastitis conditions is moderate to high in poor bedding conditions (58). *Blautia* and *Lactobacillus*, a genus with probiotic characteristics, may decrease during disease, whereas the genus *Bacillus* contains both pathogenic and probiotic species; perhaps the abundance of probiotic strains is more prevalent in healthy conditions. It is not easy to speculate and interpret until further multi-bacterial culture-based research investigation. Different *Bacillus* strains have been reported to exhibit antimicrobial, antioxidant, and immune-modulatory activities in the host. A study by Pinchuk et al. (32) has reported the *B. subtilis* strains’ anti *H. pylori* activity, which was attributed to the secretion of aninocoumacin A antibiotic. The antagonistic activity of aninocoumacin A was also documented against enteric *E. faecium* and *Shigella flexneri*. An interesting communication by Ripert et al. (59) revealed that the probiotic *B. clausii O/C* strain protected Vero and Caco-2 cells from the cytotoxic effects of *Clostridium difficili* and *B. cereus* toxins.

*Pseudomonas*, *Stenotrophomonas*, *Comamonas*, and *Sphingomonas* are among the most transcriptionally active pathogens. *Pseudomonas* and *Sphingomonas* genera are often reported in association with bovine mastitis in various studies (60, 61). The *Stenotrophomonas* genus is a lesser-known opportunistic pathogen associated with bovine mastitis. However, various *Stenotrophomonas* isolates were extracted and reported from bovine milk samples for mastitis cases (62). The Prevalence and survival of *Stenotrophomonas* species in milk and dairy products have also been reported (63, 64). A study has explored the prevalence of genetic relatedness, antimicrobial resistance, biofilm formation, biofilm genes associated with virulence and integron genes among isolates of *S. maltophilia* recovered from bovine milk with subclinical mastitis (65). *Comamonas* is another less-known genus associated with bovine mastitis. However, it was isolated from bulk tank milk (66). Therefore, our study emphasises the inclusion of these prominent pathogens in the list of primary bovine mastitis-causing pathogens.

### 4.1 Limitations

This study utilised different host-centric RNA-Seq studies to identify transcriptionally active bacteria and their molecular signature. However, these studies have their own experimental designs, sequencing methods (single-end/paired-end/different platforms), and data analysis approaches to achieve their objectives. It is very challenging to obtain datasets from the same source with identical technical specifications. Therefore, a uniform data processing approach has been used to extract and assemble the metatranscriptome to minimise confounding risks and technical disparity. Each study was processed and reported separately before being integrated into the overall analysis. A fraction of the non-host reads from host-centric dual RNA-seq studies has been extracted and used to construct a metatranscriptome, rather than a microbial-centric metatranscriptome. Additionally, transcription levels do not always correlate with protein abundance or activity. Therefore, these findings may differ slightly from the actual situation. This study aims to generate research leads for scientists by leveraging computational tools and publicly available datasets. However, *in vitro* validation of these findings is crucial for a better understanding of the microbial aspects of bovine mastitis, which is beyond the current scope of this work.

## 5 Conclusion

This is the first in-depth metatranscriptomics study on bovine mastitis, delineating transcriptionally active pathogenic bacterial taxa, their associated molecular signatures, and uncovering a complex pathogenic consortium that goes beyond the well-known causative pathogens. Pseudomonas (*P. putida, P. aeruginosa, P. fluorescens, P. monteilii, P. mosselii*, and *P. plecoglossicida*), *Stenotrophomonas* (*S. maltophilia*, and *S.* sp. *RIT309*, *SKA14*), *Mycoplasma* (*M. bovis, M. hyorhinis, M. agalactiae*, and *M. ovipneumoniae*), *Staphylococcus* (*S. aureus*, *S. succinus*, and *S. sciuri*), *Escherichia* (*E. coli CFT073*, and *NA114*), *Comamonas* (*C. aquatica*, *C. testosteroni*) and *Sphingomonas* (*S.* sp. *IBVSS2*, *LK11*, *FARSPH*, *S. sanguinis*, and *S. taxi*) species were predominant among transcriptionally active pathogens, and should be considered as significant contributors to bovine mastitis pathogenesis alongside established pathogens.

Identified 213 hypothetical and uncharacterized proteins from *Staphylococcus*, *Mycoplasma*, and *Escherichia,* providing a list of unexplored virulence factors that could serve as targets for multi-epitope vaccine development, while the expression of 74 AMR genes, particularly from *Pseudomonas* and *Escherichia* species, suggests these pathogens may confer antibiotic protection to the entire pathogenic consortium, necessitating the need to update current treatment strategies. The abundance of uncharacterized proteins presents opportunities for developing novel diagnostics and therapeutics. The negative correlation of beneficial bacteria (*Blautia*, *Bacillus*, and *Lactobacillus*) with pathogenic species indicates that microbiome modulation could be a viable prevention strategy for subclinical mastitis. A balanced microbiome with these antagonistic bacteria could help to reduce pathogen loads and prevent subclinical infections from becoming clinical. However, these findings need to be validated thoughtfully to develop microbiome-assisted prevention strategies for subclinical bovine mastitis. Our study highlights that transcriptionally active bacteria and their expressed genes (AMR, secretory, peptidase and virulence genes) can aid in identifying novel therapeutic targets, early diagnostic biomarkers and expressed candidate genes for developing a multi-epitope vaccine that targets multiple pathogens. Our findings also suggest the potential of microbiome modulation strategies to develop microbiome-assisted prevention approaches for better management of subclinical bovine mastitis.

## Data Availability

The datasets presented in this study can be found in online repositories. The names of the repository/repositories and accession number(s) can be found in the article/Supplementary material.
