# Supplementary material for: Metatranscriptome analysis to unveil the molecular signatures of transcriptionally active pathogens associated with bovine mastitis
Source: Front Vet Sci. 2025 Oct 16;12:1642351. doi: 10.3389/fvets.2025.1642351 (PMC12571623; doi:10.3389/fvets.2025.1642351)
Supplement: Supplementary file 5 [file Data_Sheet_1.docx]

**Supplementary Materials**

**Table S1:** Selected RNA-Seq studies for metatranscriptomics analysis to identify transcriptionally active microbial species associated with bovine mastitis.

| **Sr. no** | **Study title** | **Data type** | **Study type** | **Tissue** | **Project ID** |
| --- | --- | --- | --- | --- | --- |
| 1 | Bovine milk somatic cell transcriptomic response to Staphylococcus aureus is dependent on strain genotype. | mRNA | Field | Milk | PRJEB43443 |
| 2 | Genetic mechanisms regulating the host response during mastitis. | mRNA | Field | Milk | PRJNA544129 |
| 3 | Effect of two different drug-resistant *Staphylococcus aureus* strains on the physiological properties of MAC-T cells and their transcriptome analysis. | mRNA | Cell line | MACT cells | PRJNA778892 |
| 4 | Integrated analysis of transcriptome mRNA and miRNA profiles reveals self-protective mechanisms of Bovine MECs induced by LPS. | mRNA | Cell line | MACT cells | PRJNA556769 |
| 5 | RNA-Seq whole transcriptome analysis of Bovine Mammary Epithelial Cells in response to intracellular *Staphylococcus aureus.* | mRNA | Cell line | MACT cells | PRJNA591729 |
| 6 | Feeding a Saccharomyces cerevisiae fermentation product improves udder health and immune response to a Streptococcus uberis mastitis challenge in mid-lactation dairy cows. | mRNA | Field | Mammary gland, Liver | PRJNA627642 |
| 7 | Genome-wide analysis of mRNAs and lncRNAs in Mycoplasma bovis infected and non-infected bovine mammary gland tissues. | mRNA | Field | Mammary gland | PRJNA551141 |
| 8 | Whole blood transcriptome analysis during immunization and mammary challenge with E. coli (cattle) | mRNA | Field | Blood | PRJNA668296 |

**Table S2:** Experiment design and sample information of selected studies for meta-transcriptomics data analysis

| **Project sample conditions and sample count** | | | |
| --- | --- | --- | --- |
| PRJNA627642 Infection agent: *Streptococcus uberis* | | | |
| Condition 1: diet type | Condition 2: infection site | Condition 3: time post-infection | Sample Count |
| Control diet | Mammary gland | 36 hours | 8 |
|  |  | 30 days | 8 |
|  | Liver | 36 hours | 8 |
|  |  | 30 days | 8 |
| NutriTek diet | Mammary gland | 36 hours | 8 |
|  |  | 30 days | 8 |
|  | Liver | 36 hours | 8 |
|  |  | 30 days | 8 |
| PRJEB43443 Infection agent: *Staphylococcus aureus* | | | |
|  | Condition 1: Infection strain | Condition 2: time post-infection | Sample Count |
|  | *Staph.aureus* strain MOK23 | 0 hours | 5 |
|  |  | 24 hours | 5 |
|  |  | 48 hours | 5 |
|  |  | 72 hours | 5 |
|  |  | 168 hours | 5 |
|  | *Staph.aureus* strain MOK124 | 0 hours | 6 |
|  |  | 24 hours | 5 |
|  |  | 48 hours | 4 |
|  |  | 72 hours | 5 |
|  |  | 168 hours | 3 |
| PRJNA591729 Infection agent: *Staphylococcus aureus* | | | |
|  | | Condition 1: sample source | Sample Count |
|  |  | Mastitic | 3 |
|  |  | Healthy | 3 |
| PRJNA556769 Infection agent: *Escherichia coli* | | | |
|  | | Condition 1: time post-infection | Sample Count |
|  |  | 0 hours | 1 |
|  |  | 4 hours | 1 |
|  |  | 8 hours | 1 |
|  |  | 12 hours | 1 |
| PRJNA551141 Infection agent: *Mycoplasma bovis* | | | |
|  | | Condition 1: sample source | Sample Count |
|  |  | Mastitic | 2 |
|  |  | Healthy | 3 |
| PRJNA544129 Infection agent: Naturally infected | | | |
|  | | Condition 1: sample source | Sample Count |
|  |  | Mastitic | 36 |
|  |  | Healthy | 37 |
| PRJNA778892 Infection agent: *Staphylococcus aureus* | | | |
|  | | Condition 1: Infection strain | Sample Count |
|  |  | *Staph.aureus* strain MSSA | 3 |
|  |  | *Staph.aureus* strain MRSA | 3 |
|  |  | Uninfected | 3 |

**Table S3:** Project-wise average abundance of pathogens by using identified distinct sequences.

| **Bacterial organisms with the most abundance in each project** | | | | | |
| --- | --- | --- | --- | --- | --- |
| **Organism** | **Abundance** | | **Organism** | **Abundance** | |
|  | **Healthy** | **Mastitic** |  | **Healthy** | **Mastitic** |
| PRJNA591729 | | | PRJNA556769 | | |
| *Mycoplasma bovis CQ-W70* | 2.7 | 3.3 | *Mycoplasmopsis arginini* | 3.9 | 2.9 |
| *Enterococcus faecalis* | 7.9 | 3.4 | *Bacillus sp. VT-16-64* | 3.1 | 3.1 |
| *Mycoplasma* | 12.7 | 14.7 | *Chlamydia trachomatis* | 1.6 | 3.8 |
| *Mycoplasma bovis 8790* | 23.9 | 16.9 | *Acinetobacter baumannii* | 4.7 | 4.6 |
| *Staphylococcus aureus* | 103.6 | 30.1 | *Klebsiella pneumoniae* | 7.3 | 4.8 |
| *Mycoplasma bovis 1067* | 72.0 | 100.8 | *Nocardioides sp. BN130099* | 12.6 | 7.4 |
| *Mycoplasma bovis ATCC 25523* | 1056.0 | 807.8 | *Proteobacteria* | 9.4 | 9.8 |
| *Mycoplasma alkalescens* | 1903.4 | 845.0 | *Salmonella enterica* | 11.0 | 11.4 |
| *Mycoplasma bovis* | 2324.9 | 1661.3 | *Staphylococcus aureus* | 17.4 | 22.9 |
| *Mycoplasma agalactiae* | 39097.7 | 25254.7 | *Anaplasma phagocytophilum* | 65.4 | 80.7 |
| PRJNA551141 | | | PRJEB43443 | | |
| *unclassified Flavobacterium* | 1.4 | 7.6 | *Klebsiella pneumoniae* | 3.9 | 3.9 |
| *Anaplasma phagocytophilum* | 6.6 | 9.7 | *Enterococcus faecalis* | 3.2 | 5.1 |
| *Mycobacteriaceae bacterium 1482268.1* | 9.7 | 9.9 | *Eggerthia catenaformis* | 1.6 | 5.9 |
| *Pseudomonas sp. SO-A5-26* | 20.3 | 12.6 | *Streptococcus pneumoniae* | 13.7 | 10.2 |
| *Marivirga lumbricoides* | 6.9 | 14.3 | *Enterococcus faecium* | 4.2 | 11.2 |
| *Campylobacter jejuni 81-176* | 17.6 | 23.9 | *Idiomarina aestuarii* | 27.7 | 21.6 |
| *Mycoplasma hyorhinis* | 59.2 | 33.9 | *Anaplasma phagocytophilum* | 10.1 | 28.3 |
| *Staphylococcus aureus* | 404.1 | 177.8 | *Mycobacteriaceae bacterium 1482268.1* | 2371.8 | 39.7 |
| *Flavobacteriaceae bacterium BH-SD17* | 9929.9 | 16567.9 | *Staphylococcus aureus* | 12.5 | 42.4 |
| *Vibrio cholerae* | 30657.4 | 34579.4 | *Xanthomonas oryzae* | 1083.1 | 232.7 |
| PRJNA544129 | | | PRJNA778892 | | |
| *Meiothermus silvanus ATCC 700542* | 1.6 | 1.1 | *Mycoplasma hyorhinis HUB-1* | 2.2 | 4.0 |
| *Bacillus obstructivus* | 29.8 | 2.2 | *Tissierella creatinini* | 1.3 | 6.0 |
| *Anaplasma phagocytophilum* | 8.2 | 11.5 | *Chlamydia* | 1.3 | 7.6 |
| *Blautia luti* | 157.4 | 12.2 | *Mycoplasma hyorhinis* | 19.7 | 7.6 |
| *Enterobacter cloacae* | 267.0 | 13.9 | *Capnocytophaga leadbetteri* | 5.6 | 8.7 |
| *Bacillus cereus R309803* | 328.3 | 16.1 | *Mycoplasma hyorhinis SK76* | 3.3 | 8.8 |
| *Porphyromonas macacae* | 438.9 | 60.1 | *Caulobacterales* | 86.1 | 18.2 |
| *Staphylococcus aureus* | 226.1 | 291.1 | *Escherichia coli* | 1.5 | 22.0 |
|  |  |  | *Anaplasma phagocytophilum* | 125.6 | 63.8 |
|  |  |  | *Staphylococcus aureus* | 362.6 | 182.3 |

**Table S4.** Sequence annotation of hypothetical/uncharacterised proteins identified from each project. Hypothetical proteins which have at least two occurrences in the mastitic samples are shown in the table and considered for further analysis.

| **Project** | **PRJNA**  **591729** | **PRJNA**  **778892** | **PRJNA**  **551141** | **PRJEB**  **43443** | **PRJNA**  **556769** | **PRJNA**  **544129** |
| --- | --- | --- | --- | --- | --- | --- |
| Hypo/Uncha | 72 | 44 | 780 | 302 | 22 | 16 |
| MobiDBLite | 38 | 12 | 40 | 15 | 8 | 5 |
| Coils | 13 | 5 | 10 | 9 | 2 | 1 |
| Panther | Craniofacial development protein-2 (3); DNA polymerase from transposon bs-like protein (3) | Line-1 RTE orf1 protein (3); L1 transposable element-related (2) | Mechanosensitive channel msck (2) | DNA polymerase from transposon bs-like protein (13); Bucentaur related (4) ; Line-1 RTE orf1 protein: 2; RT (6) | Bucentaur-related (1); DNA polymerase from transposon bs-like protein-related (2) | Craniofacial development protein-2 (2); Bucentaur related (1); RT(1); DNA polymerase from transposon bs-like protein-related (1) |
| Superfamily | DNase I-like: 3 | Mss4-like: 1; Acyl-CoA N-acyltransferases (1); DNA/RNA polymerases: 1; DNase I-like: 2 | DNA/RNA polymerase (1); Adhesin YadA, collagen-binding domain (2); Porins (2) | P-loop containing nucleoside triphosphate hydrolases: 2 | DNase I-like: 4 | DNase I-like: 1 |
| Print | - | Translationally controlled tumour protein signature ( 3) | Glucose/ribitol dehydrogenase family signature ( 8); Short-chain dehydrogenase/reductase signature (3) ; Pi haemoglobin signature: 4; α haemoglobin signature (5) | - | - | - |
| Pfam | Endonuclease/Exonuclease/phosphatase family (2) | L1 transposable element RBD-like domain (2) | Coiled stalk of trimeric autotransporter adhesin (2) | L1-TE trimerization domain (2); L1-TE RBD-like domain (3) | RT( 1); Endonuclease-reverse transcriptase (1) | Endonuclease/Exonuclease/phosphatase family: 1 |
| Prosite | RT catalytic domain profile (1); PML-lipid attachment site profile (3) | PML- lipid attachment site profile (1) | - | - | RT catalytic domain profile (1) | - |
| CDD | Endonuclease domain of the non-LTR retrotransposon LINE-1 and related domains: 2 | RT_nLTR_like and  L1-EN domain: 1 | - | - | Endonuclease domain of the non-LTR retrotransposon LINE-1 and related domains: 2 | Endonuclease domain of the non-LTR retrotransposon LINE-1 and related domains: 1 |

TE: transposable element; RTE: Retrotransposable element; RT: Reverse transcriptase; PML: Prokaryotic membrane lipoprotein

**Table S5.** Description of KO enriched pathways and counts of mapped KO terms

| **Pathways Description** | **Count** |
| --- | --- |
| Biosynthesis of amino acids | 116 |
| Biosynthesis of cofactors | 129 |
| Two-component system | 154 |
| Carbon metabolism | 107 |
| Glycine, serine and threonine metabolism | 47 |
| Pyruvate metabolism | 48 |
| Flagellar assembly | 32 |
| Alanine, aspartate and glutamate metabolism | 35 |
| Bacterial secretion system | 36 |
| Bacterial chemotaxis | 20 |
| Purine metabolism | 64 |
| Biofilm formation - Pseudomonas aeruginosa | 37 |
| Glyoxylate and dicarboxylate metabolism | 39 |
| Arginine and proline metabolism | 40 |
| Cysteine and methionine metabolism | 42 |

A)


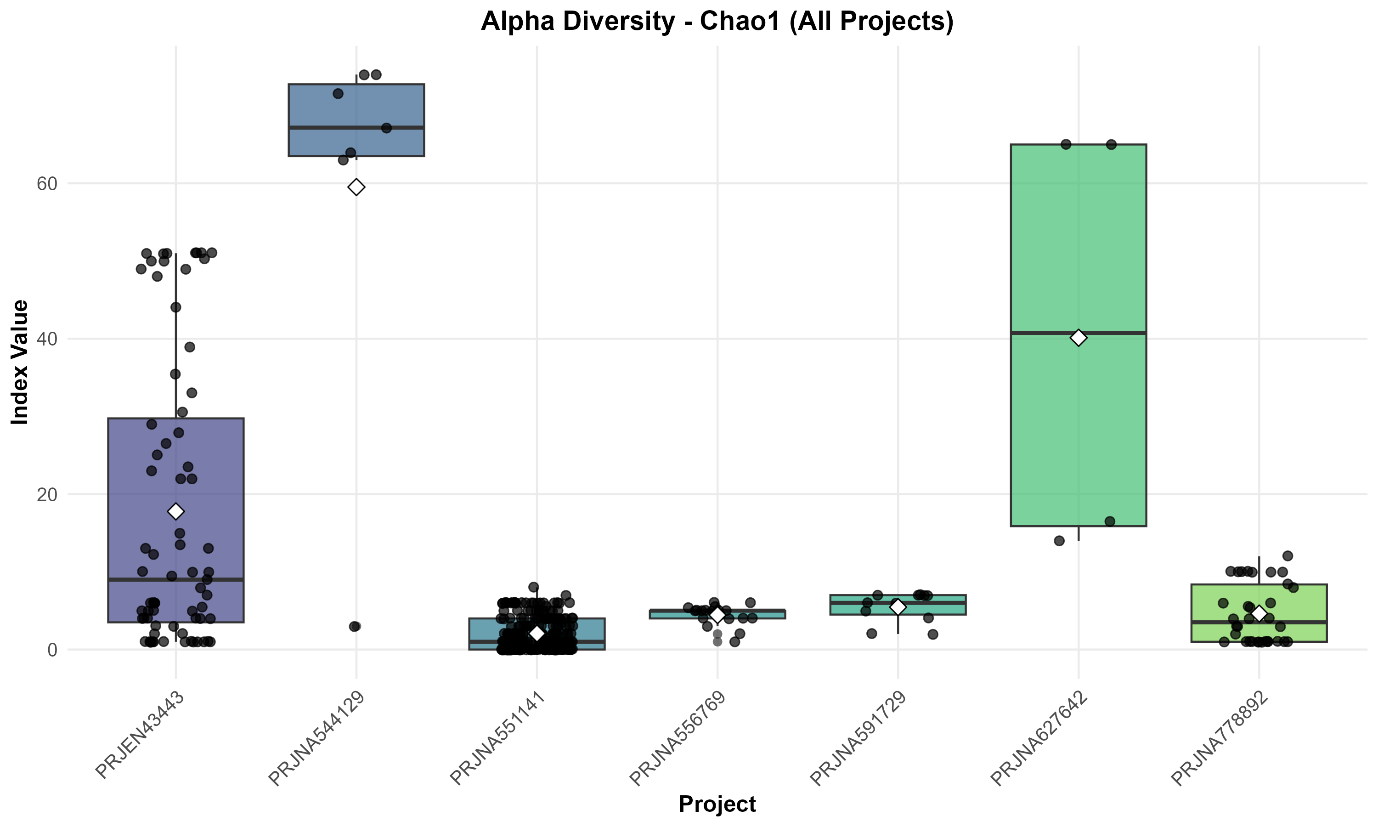


B)


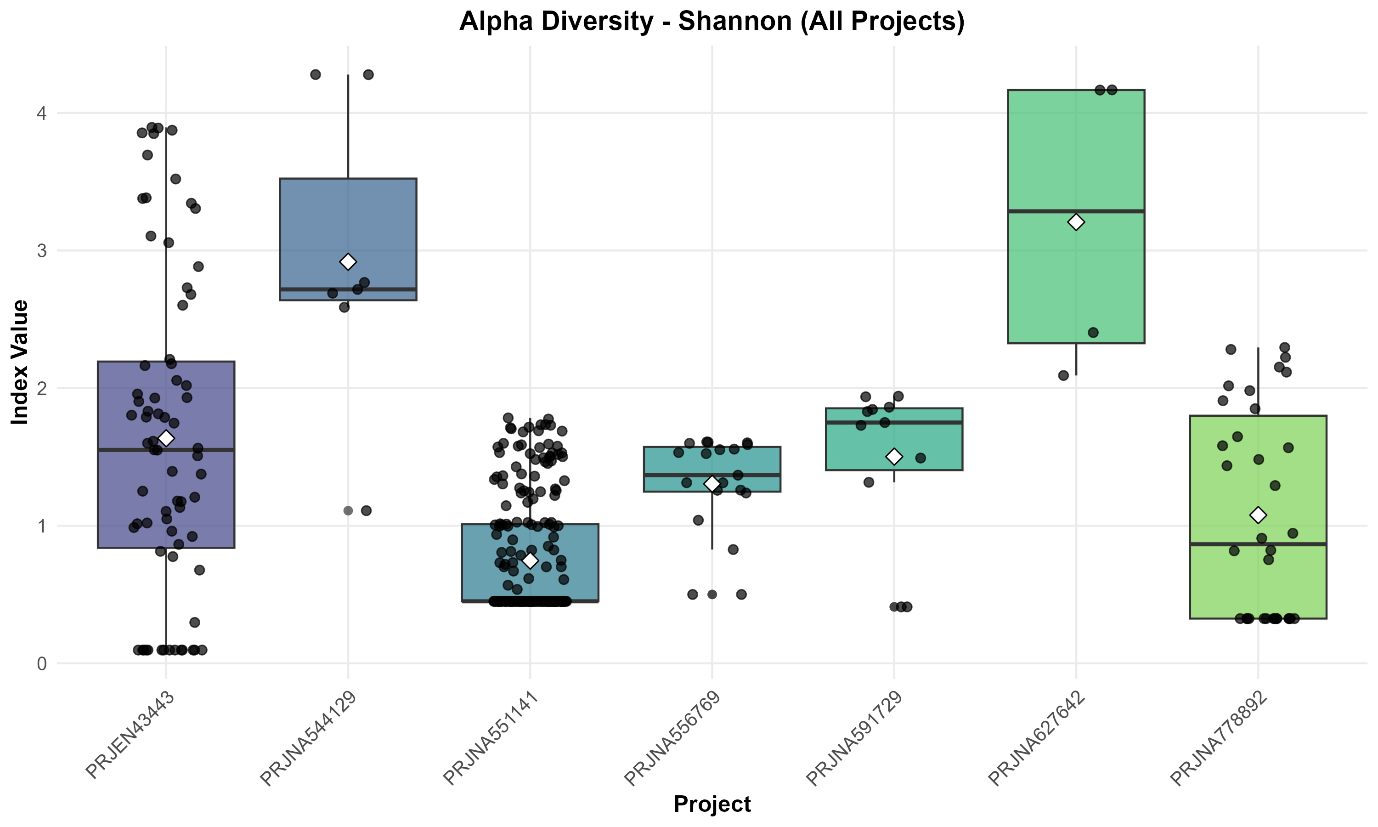


C)


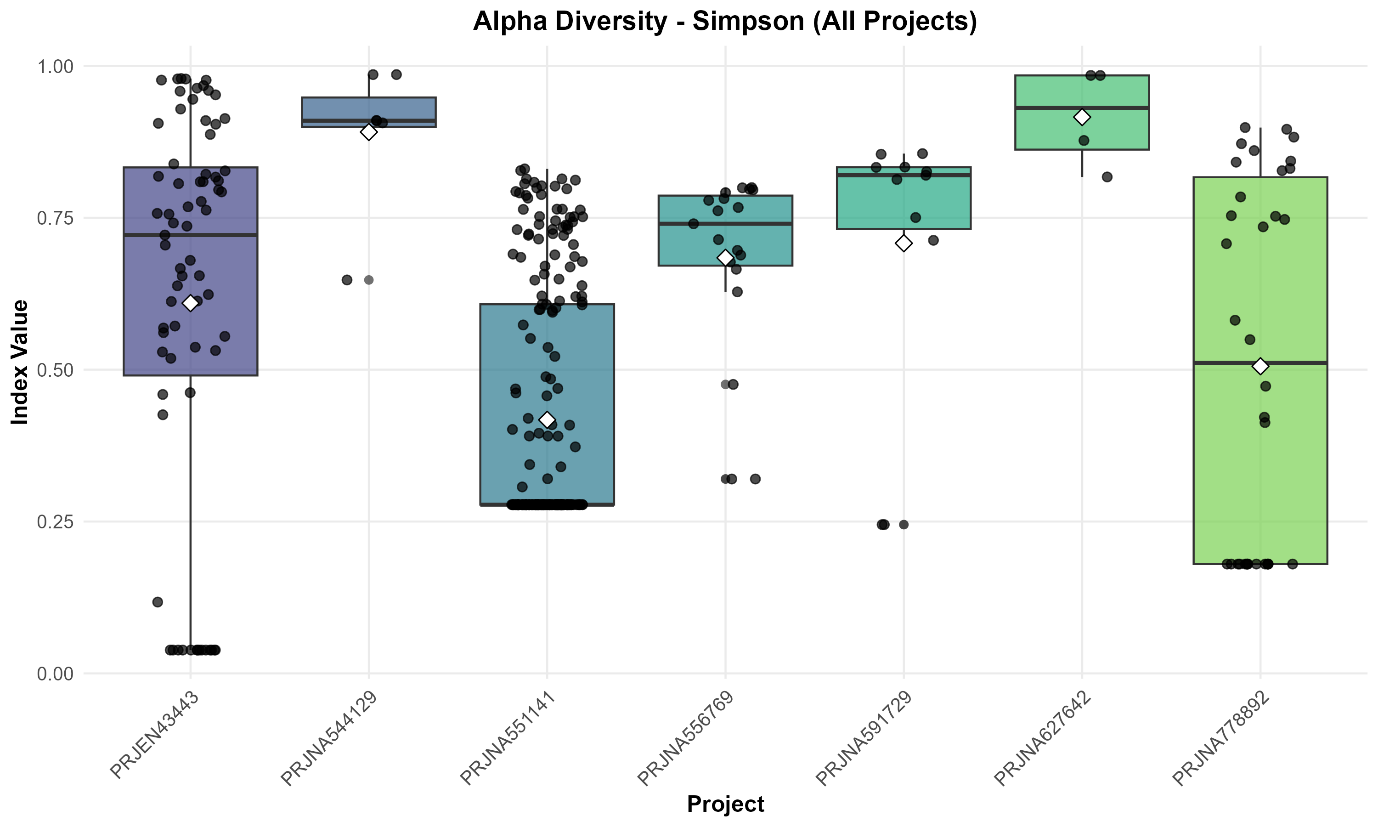


D)


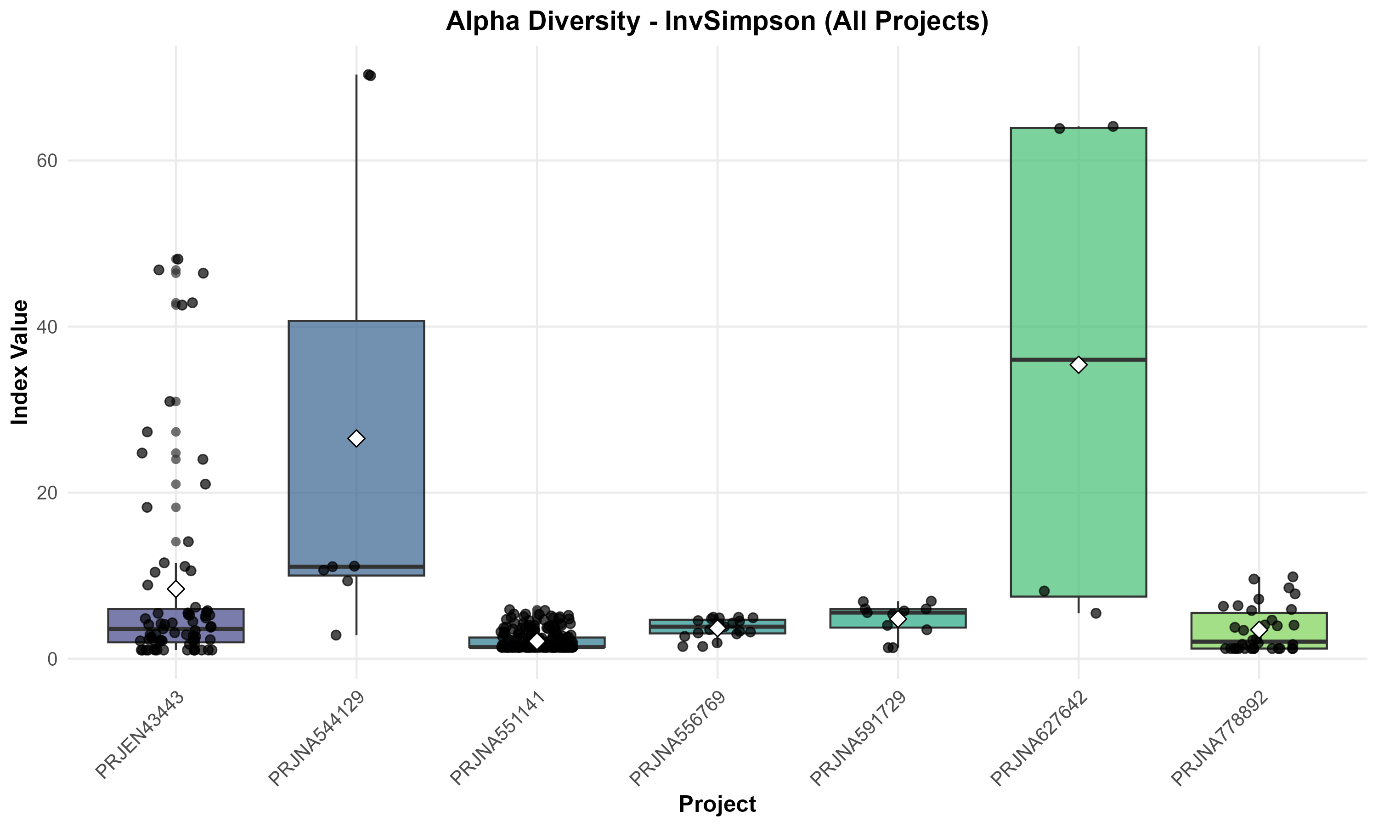


E)


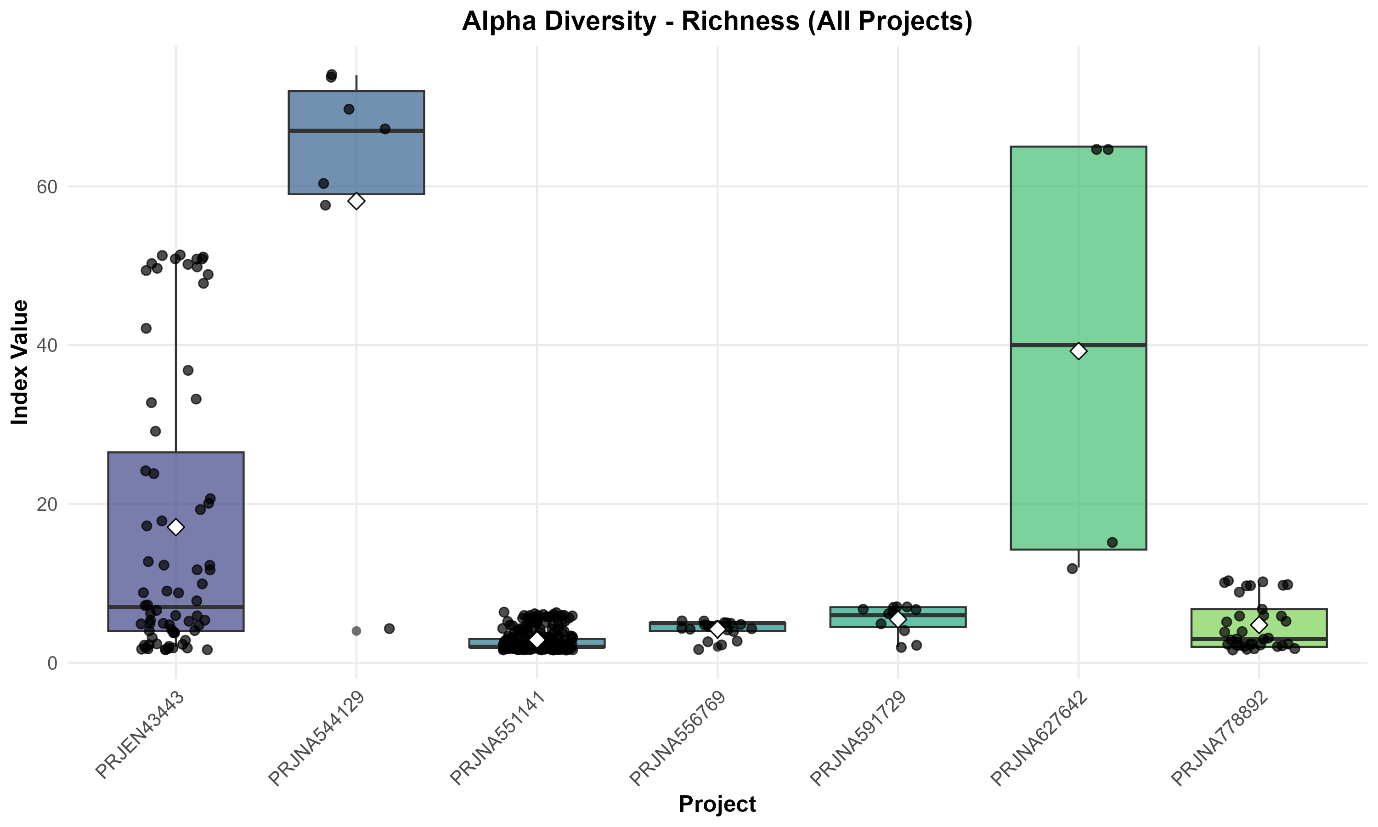


Figure S1. Different diversity indices: A) Chao1, B) Shannon, C) Simpson, D) InvSimpson, and E) Richness.


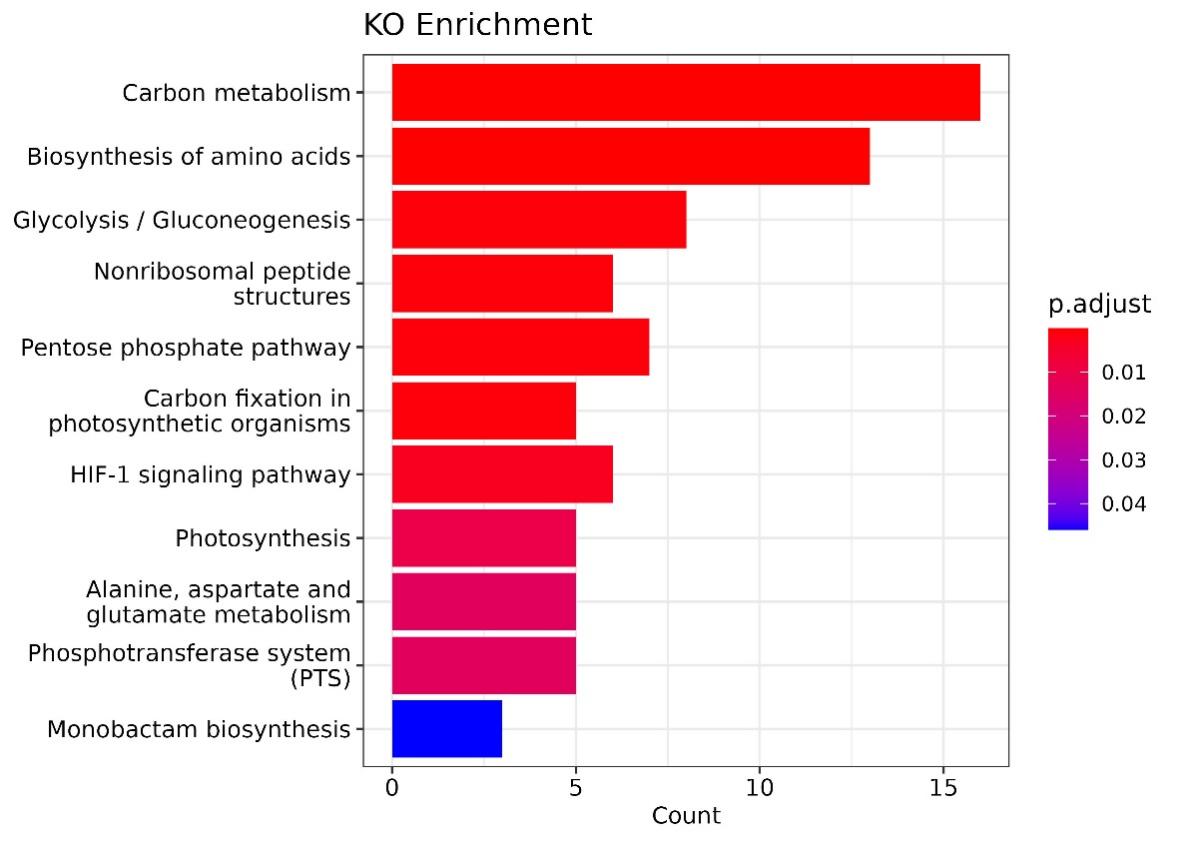


Figure S2(a). KO enrichment of PRJEB43443


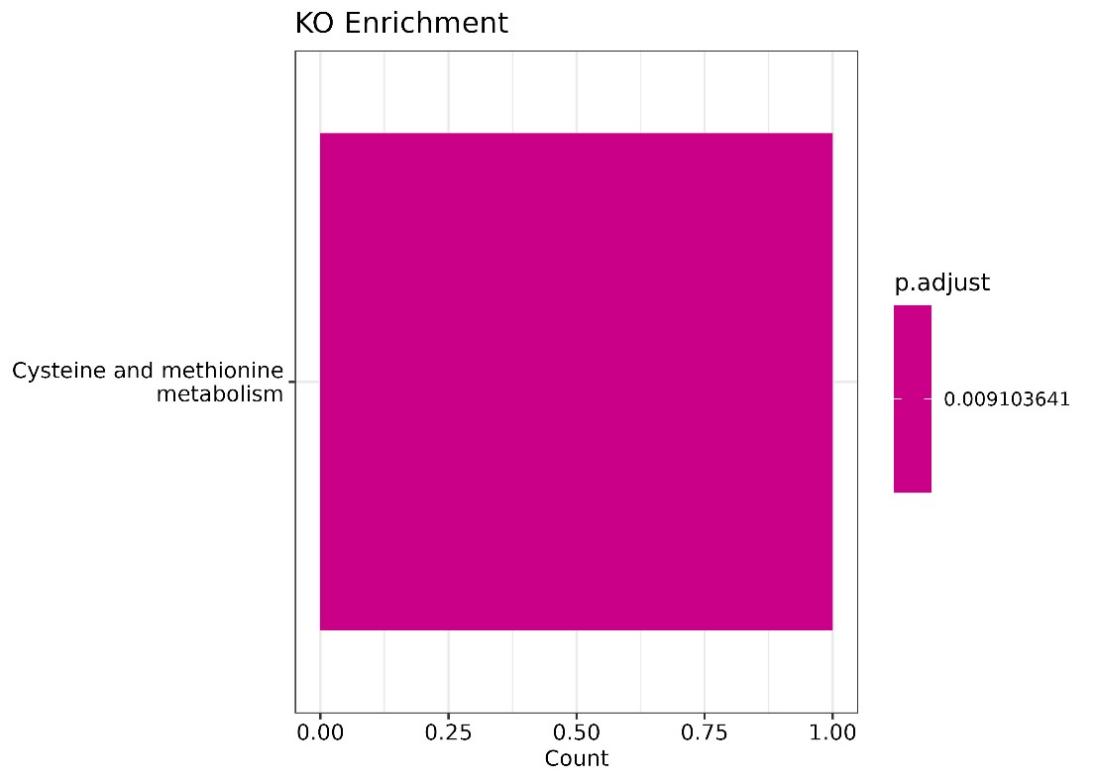


Figure S2(b). KO enrichment of PRJNA544129


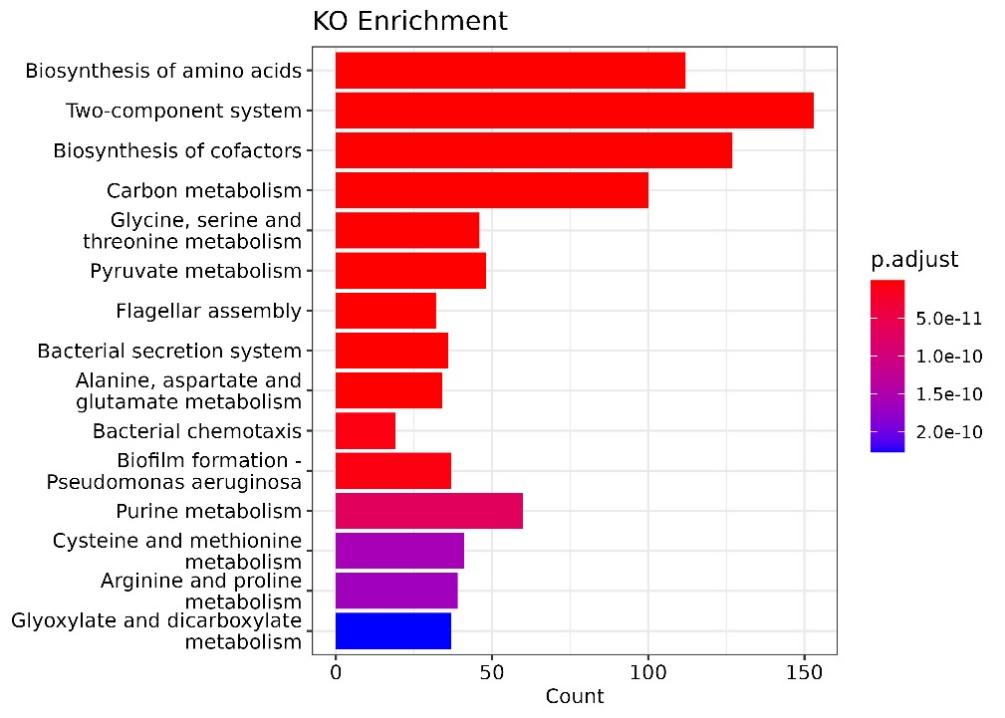


Figure S2(c). KO enrichment of PRJNA551141


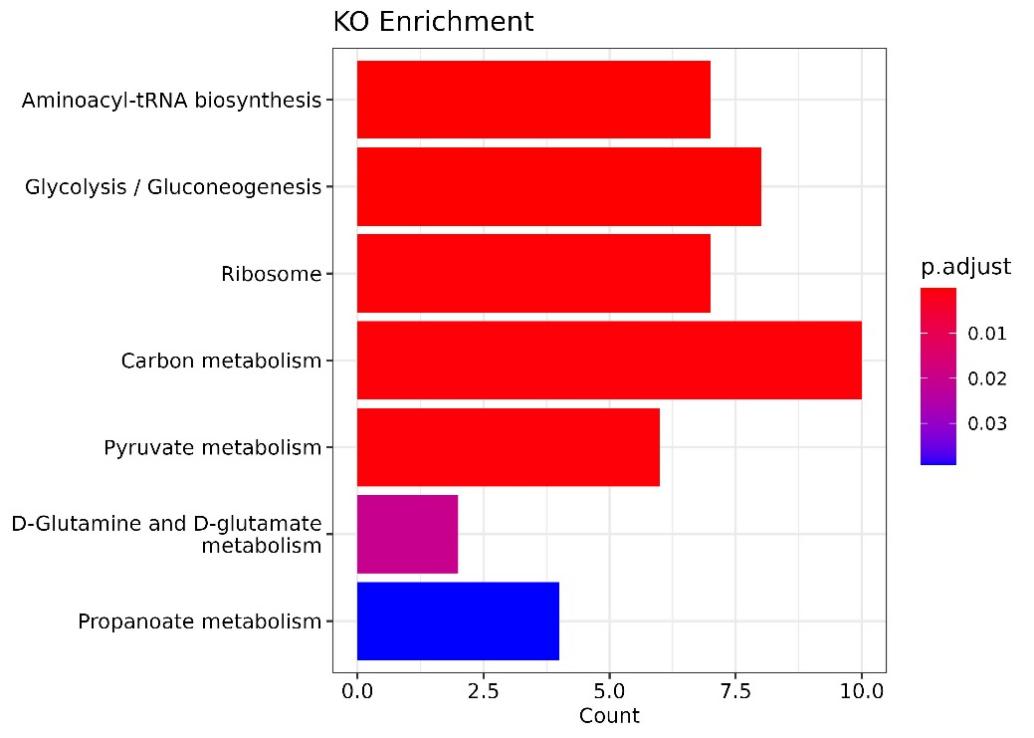


Figure S2(d). KO enrichment of PRJNA778892


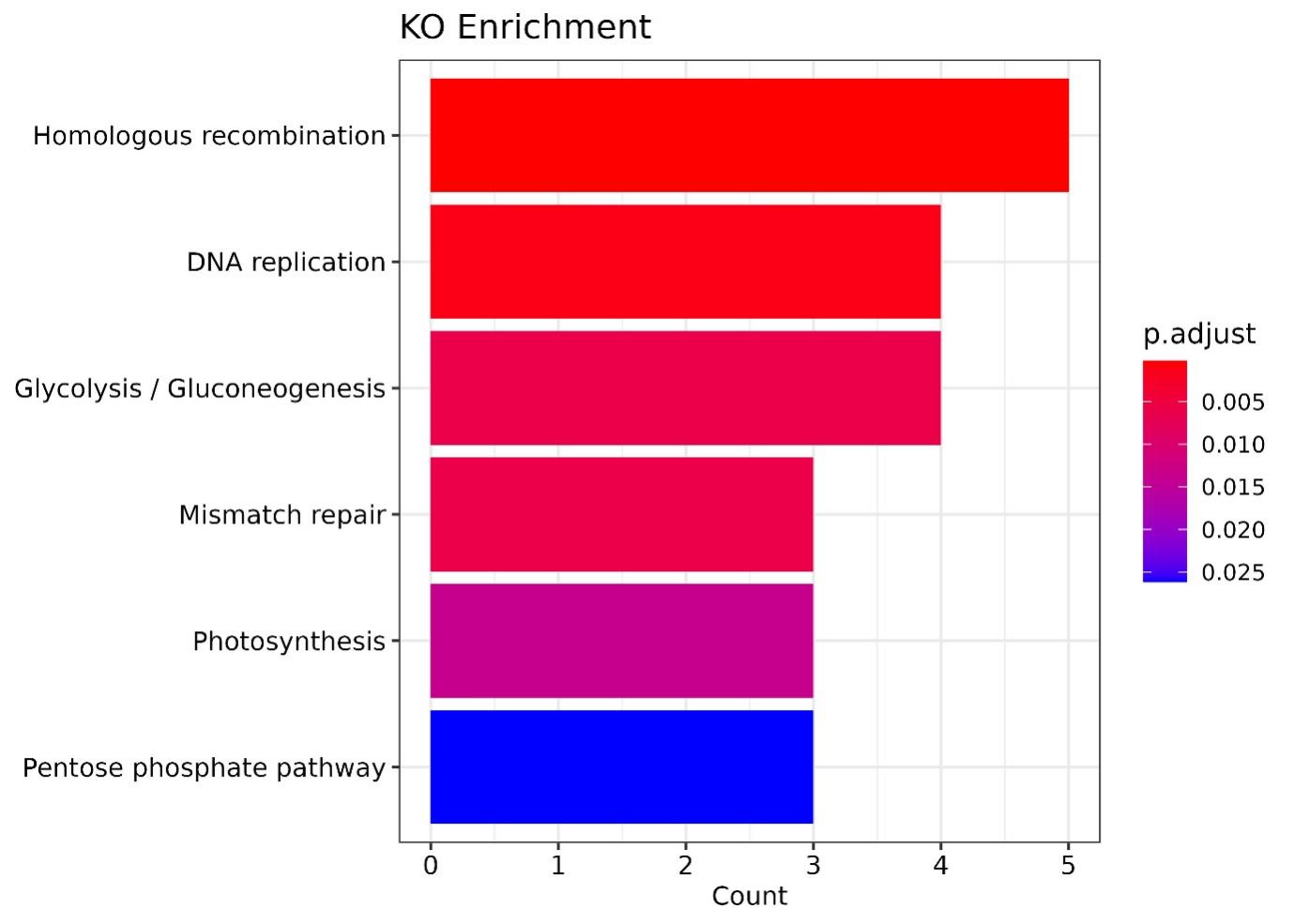


Figure S2(e). KO enrichment of PRJNA591729
